# Emerging Trends in Machine Learning: A Polymer Perspective

**DOI:** 10.1021/acspolymersau.2c00053

**Published:** 2023-01-18

**Authors:** Tyler B. Martin, Debra J. Audus

**Affiliations:** National Institute of Standards and Technology, Gaithersburg, Maryland20899, United States

**Keywords:** Polymers, Machine Learning, Artificial Intelligence, Autonomous Experimentation, Transfer Learning, Explainability, Optimization, Inverse Design, Deep Learning, Open Science

## Abstract

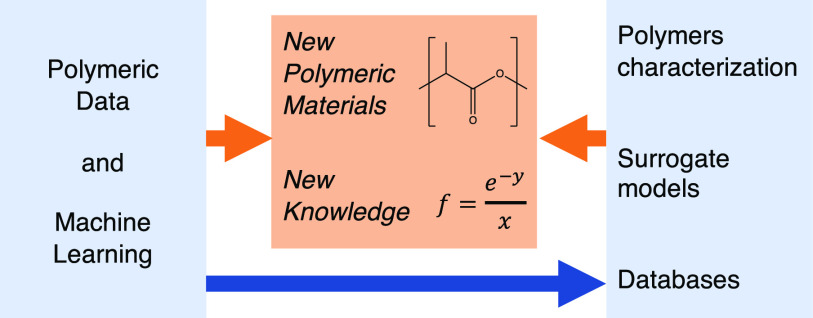

In the last five years, there has been tremendous growth in machine learning and artificial intelligence as applied to polymer science. Here, we highlight the unique challenges presented by polymers and how the field is addressing them. We focus on emerging trends with an emphasis on topics that have received less attention in the review literature. Finally, we provide an outlook for the field, outline important growth areas in machine learning and artificial intelligence for polymer science and discuss important advances from the greater material science community.

## Introduction

Artificial intelligence (AI) has already revolutionized our daily lives from self-driving cars to semantic language translation to tailored content feeds and beyond. The latest AI image generation models can transform text strings into images that can be nearly indistinguishable from high-quality human generated art and photography.^1^ In medicine, machine learning (ML) models are being used to identify carcinogens and diagnose diseases such as Parkinson’s that, previously, could not be identified from biomarkers.^2,3^ Decades long scientific problems, such as the classic “protein folding” problem, are being tackled by AI that produce results which approach the resolution of our best measurements.^4^ With each major advance, it is clear that we have yet to realize the full impact of AI and ML.

Even within the materials science community, the application of ML and AI techniques is becoming routine.^5−8^ For the purposes of this article, ML is the use of mathematical models to perform well-defined data tasks such as clustering, classification, or regression. AI is a more difficult term to define, but generally refers to the emergent “behaviors” that arise from complex stacks of ML and data models. Given that these terms are often used interchangeably, for simplicity we will refer to ML rather than AI and ML in this article. Over the past five years or so, there has been tremendous progress in the application of these methods to polymer problems as detailed in numerous perspectives and reviews.^9−20^ Polymers focused researchers are using ML to accelerate the discovery of new materials and new knowledge, as well as working to overcome barriers such as data scarcity. For example, ML has enabled the generation of potential new polymer chemistries,^21^ new materials for gas separation membranes,^22^ prediction of properties for sequence defined polymers,^23^ bioplastic design,^24^ guidance for improving 3D printing,^25^ improved contrast agents for magnetic resonance imaging (MRI) measurements,^26^ and methods for improved predictions of very small data sets.^27^

Despite this progress, the field is still plagued by a variety of challenges that arise from both the unique and nonunique problems associated with polymer science. Unlike many kinds of materials, the structure of polymers is inherently stochastic rather than a single structure. This makes the representation of polymers in ML models a challenge. Furthermore, “big data” ML (i.e., data set sizes close to a billion) is currently out of reach for the polymer community as there are no publicly available databases that provide enough well-tagged polymer data to support such an endeavor. Many of the key measurements leveraged in the polymer community rely on instruments made by manufacturers that do not provide open interfaces and data models for their devices, impeding the creation of databases and making the integration of these devices into high-throughput and automation platforms nearly impossible. Table 1 expands upon the current list of challenges facing the polymers community and categorizes them into broad areas.

**Table 1:** Challenges Facing the Polymer Machine Learning Community

category	challenge	paper section
polymer nature	polymer structure is stochastic and hierarchical	Data, Polymer Representations, Deep Learning
polymer nature	morphology is process history dependent	Data, Domain Knowledge, Optimization and Inverse Design
polymer nature, community	data is not produced in standardized formats	Data, Polymer Representations, Unsupervised Analysis
community	(meta)data is not complete, accessible, or shared	Data, Unsupervised Analysis, Open Science
community	code is not accessible or open	Open Science
community	available data is small and disperse	Data, Data Fusion and Transfer Learning, Domain Knowledge, Open Science, Beyond Polymer Autonomous
community	analyses are not reproducible	Open ScienceBest Practices and Challenge Problems
community	models do not provide uncertainty quantification	Data, Autonomous Experimentation, Data Fusion and Transfer Learning, Best Practices and Challenge Problems
community	models are not explainable	Interpretability and Explainability, Domain Knowledge, Unsupervised Analysis
community	models do not extrapolate	Domain Knowledge
hardware	custom hardware is hard to use and adapt outside of initial study	Autonomous Experimentation
hardware	commercial hardware has poorly documented or closed interfaces	Beyond Polymer Autonomous
all	large combination of skills needed to carry out studies	Data, Autonomous Experimentation, Beyond Polymer Autonomous

In this paper, we first give an overview in Creating an ML Pipeline and then provide Updates on two of the largest challenges that continue to plague the polymers community: Data and Polymer Representations. Next, we highlight growth areas, many of which have received less focus in recent polymer reviews, in New Progress. Since polymer ML is a rapidly growing field, especially since 2017, we focus on recent studies and limit discussion on topics that are well-covered in recent reviews, such as the application of ML to simulations (see refs (11) and (28)) and inverse design (see ref (15)). Finally, we conclude with an Outlook section that provides an editorial assessment on several of the areas of new progress and discusses important, but less discussed topics including the role of Open Science and Best Practices and Challenge Problems within the ML space. To further guide the reader, we have provided key connections between the outstanding challenges and how the polymer community is addressing these challenges as categorized by paper subsections in Table 1.

## Creating an ML Pipeline

**Figure 1:**
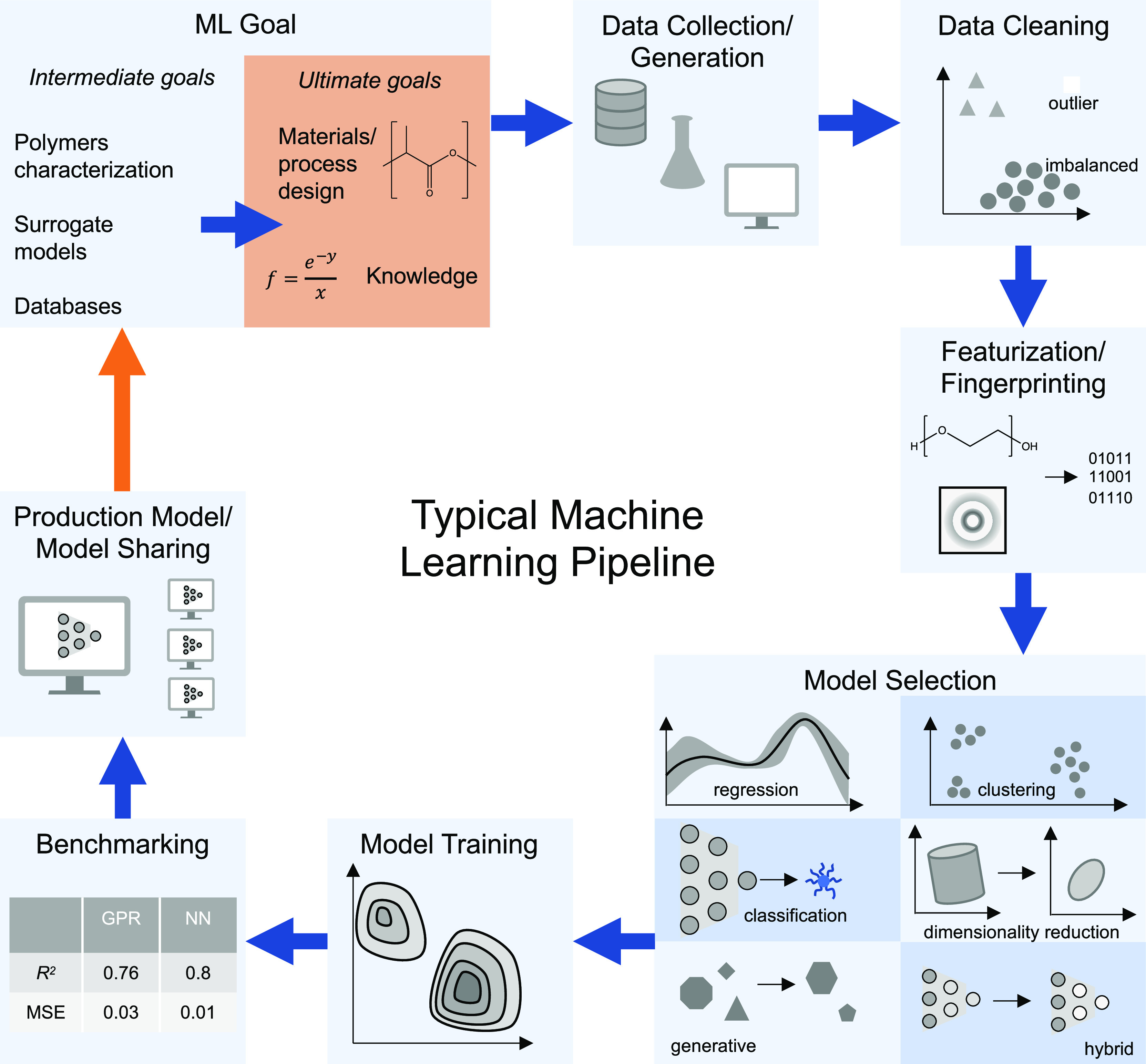
Typical pipeline for polymer ML. We emphasize that due to the rapidly growing field of ML, this pipeline does not cover all potential use cases. Acronyms include Gaussian process regression (GPR), neural network (NN), mean squared error (MSE).

Here, we provide a brief overview of the steps from conceptualization to a production ML model as shown in Figure 1. We emphasize that these steps are often a simplification of the complicated pipelines that are currently being constructed and used in production environments and that we only seek to provide a broad overview for the uninitiated reader. We direct the reader to other resources for more complete treatments of ML and model building.^29−32^

The first and most important step in developing a production ML model is problem identification. In polymer science, the there are typically two ultimate goals for ML: materials/process design and knowledge discovery. For materials/process design, example goals could be a new polymer chemistry, a new processing protocol or a new formulation. These challenges often fall under the umbrella of inverse design and normally involve property optimization. For example, selecting the polymer with the highest thermal conductivity^33^ or balancing multiple objectives.^34^ For the goal of knowledge discovery, an example could be what processing parameters are essential for a given application. However, a specific ML pipeline might have an intermediate goal such as polymer characterization (including property prediction), generating a fast surrogate model (such as replacing time-consuming experiments or simulations) or generating a database (such as a list of possible polymers or extraction of data from the literature).

The second step is data collection, generation, and selection. This could involve running new experiments or simulations, taking data from handbooks (online or otherwise), using other historical data or a combination thereof. Since ML is a data-driven technique, data selection and data quality are particularly important. Missing metadata or improperly collected data can influence models in ways that are difficult to identify and diagnose. For many polymer applications, processing history plays an essential role and this information must be captured in the metadata for ML models to be effective. At this step, if applicable, it is recommended to consider the uncertainty and determine the intrinsic error in the data set as an ML model cannot make predictions at a higher accuracy then the original data set unless additional knowledge is encoded in the model. Next, the data may need to be cleaned. This involves everything from identifying biases in the data set (such as certain values being more likely than others) to finding outliers (which could be either erroneous or interesting data) to normalizing the data.

The fourth step is featurization. This includes converting chemistry into machine readable quantities (i.e., features), a process known as fingerprinting. This can be done with hand-crafted features or using ML techniques to automatically perform the featurization. Examples of featurization include images being fed into convolutional neural networks (where the convolutional layers automatically featurize the data) or encoding the chemistry and bond connectivity of molecules in graph neural networks. At this stage, it is also advisable to identify correlations between features and determine if fewer features can be used especially if there is data scarcity.

Next is model selection. There are a variety of ML models crafted for different tasks. For example, in regression, which can be used for property prediction, a continuous output, such as density, is predicted as a function of an input, such as chemistry. Classification is similar, but the output is a discrete class such as phase separated versus homogeneous. Both of these tasks are considered supervised since the training data is labeled with an output (e.g., the density value or homogeneous/separated class). Clustering is used to group data together and can be used to identify different phases even if the type of phase is unknown. Dimensionality reduction can be used to generate knowledge by projecting complicated, high dimensional data onto a lower dimensional space that may be easier to interpret. Clustering and dimensionality reduction are considered unsupervised when the data is unlabeled (e.g., the categories in clustering are not known a priori). Generative models are designed to generate new data from existing data, such as new polymer structures from a list of previously synthesized polymers. As is becoming increasingly common, hybrid models are used where multiple ML models are combined. This can be relatively straightforward, such as performing dimensionality reduction on the features prior to another task in order to improve performance. Alternatively, it can be more complicated and integrated with other tasks such as optimization. Independent of the chosen task, key aspects in model choice are simplicity, uncertainty quantification, and performance.

After model selection is model training. This includes separating the training data into batches for separate training, testing, and cross-validation. In also includes optimizing the hyperparameters of the model. This step is particularly important because bad choices of hyperparameters can lead to models with suboptimal predictions and additionally lead to difficulty in the benchmarking step. Success in optimization may depend on the algorithm for optimization, the optimizer parameters and the quantity being optimized (e.g., minimizing mean squared error).

Benchmarking is particularly important as many aspects of ML (similar to numerics) are still an art form rather than a science. Since model training can be time-consuming, prototyping is highly recommended. It is usually useful to compare more complicated models with simpler models to determine if the additional complexity is helpful or not. Benchmarking could include comparing different model types, different hyperparameters, different data sets, different featurization schemes, etc. Often it is done by comparing error metrics such as mean squared error, *R*^2^, or, in the case of classification, *F*_1_ score. At this stage, visualization of the results is also recommended since few error metrics capture a full picture of the performance. Concerns might include extrapolation or if there are classes that are more accurate than others. For visualization, parity plots can be useful.

Finally, there is the production model. At this point, the model can be used for its intended purpose (e.g., materials/process design or knowledge discovery). Many model varieties are much faster to execute than they are to train and therefore can be applied repeatedly, or in real time after training. At this point, we highly encourage readers to share their models, benchmarking, generating code and data. As discussed in Open Science, this will ultimately further the two key goals of ML for polymers: acceleration of new materials discovery and new science.

While the above steps describe many ML models in polymer science, ML is a growing field that can defy categorization. Pipelines can become much more complicated by not only combining different ML models as previously discussed, but also by combining data from different sources as discussed in Data Fusion and Transfer Learning and by active learning, a technique where new data is selected iteratively and the ML model is updated. This framework will be discussed in more detail in Autonomous Experimentation.

## Updates

### Data

ML, by definition, relies on data. As shown in Table 1, ML for polymers has many data challenges. First, there is the issue of not having enough data. Large ML models such as Megatron-Turing Natural Language Generation,^35^ an advanced language model, and AlphaFold2,^4^ an accurate predictor of protein structure, rely on enormous data corpora covering billions to hundreds of billions of words or protein sequences.^36^ Second, there is the issue of not having enough *quality* data. For example, the glass transition temperature is an important property where there are several data sets, and yet even combining curated data sets can yield large uncertainties. Jha et al. explicitly explored this by combining three curated data sets (two handbooks and one online resource) and found that the intrinsic uncertainty was around 40 K,^37^ which is likely prohibitively large for use in polymer design. They found that using predictions of the median yielded uncertainties roughly similar to the intrinsic or irreducible uncertainty. Thus, the only way to improve the model further is to improve the data. This case study highlights that the issue of data quality is a subtle one and intricately intertwined with the issue of metadata, the contextual information for the data. Polymers are particularly complicated because (1) they are intrinsically stochastic in nature—composed not of a single molecule type, but an ensemble of different structures, (2) their properties can significantly depend on their processing history, (3) measurements can often depend on instrument settings, and (4) uncertainty quantification in both the data and metadata is often critical. Thus, it is essential to capture and ultimately use both the data and metadata in ML pipelines. Proprietary and nonstandardized data formats further exacerbate these issues. A list of different methods for obtaining data and considerations is shown in Table 2. Note that these considerations are directly related to the aforementioned challenges.

**Table 2:** Methods for Obtaining Data and Corresponding Considerations

method	considerations
manual	very limited data set sizes
high-throughput experiments	need custom hardware
	need specialized skill set
high-throughput simulations	need specialized skill set
natural language processing	data can be heterogeneous
	need specialized skill set
	metadata may not be available
	uncertainty may not be available
curated databases	limited data set sizes
	data can be heterogeneous
	metadata may not be available
	uncertainty may not be available
	data collection may be manual (no API)
user populated databases	data can be heterogeneous
	metadata may not be available
	uncertainty may not be available

To tackle these data and metadata related issues, which are ultimately issues associated with making data Findable, Accessible, Interoperable and Reusable (FAIR),^38^ there are several options. First, there are painstakingly curated handbooks and online resources, many of which include relevant metadata. However, the data set sizes are fixed and the sources can be varied resulting in heterogeneous data. Refer to Table 1 of refs (12), (13), and (14) for useful lists of such resources.

Another option is high-throughput experiments.^39^ This idea is not a new one as detailed in a recent comment^16^ and has the benefit of incorporating the relevant metadata from the start by ensuring that experiments are performed consistently. However, it cannot be broadly applied as some systems and measurements are unsuited for such experiments due to long measurement times or difficult to automate material processing steps. Furthermore, the development of high-throughput platforms can be prohibitively costly in time, money, and resources. Despite this, many researchers have pursued the development of high-throughput techniques and one branch of this field will be discussed in the Autonomous Experimentation section below.

There are also high-throughput simulations,^40−45^ which face the same benefits and challenges as high-throughput experiments with the notable differences that data and metadata from simulations are intrinsically machine readable and that simulations are often not quantitatively predictive of experiments. Thus, for polymer design, simulations are used to identify potential candidates^41^ or in the case of property predictions, experimental and simulation data must be merged or otherwise used.^42,43^

An orthogonal approach is to use ML itself to find polymer data that is published in the literature, an approach known as natural language processing (NLP). There has been some promising progress in this area notably in identifying polymer names,^46^ recognizing that the same polymer is referred to by different names,^47^ developing pipelines for property extraction,^48,49^ and generating knowledge via word embeddings, which represent words as vectors.^50^ However, the issue of deciphering the polymer name and capturing all of the relevant metadata is still not fully solved. Nonetheless, it is a promising area. For example, Lin et al. developed PolyName2Structure, which takes in polymer names (common, source, or structure) and then predicts monomers, predicts reactions, and simulates those reactions in order to yield a polymer structure.^51^ Progress in the broader materials domain^6^ shows that NLP may be a promising approach to not only get materials data, but also materials knowledge. However, for the average polymer ML developer, the skill set required to use NLP is likely prohibitive, especially since NLP suffers from many of the same problems as manually curated data sets from a data user perspective.

Finally, another approach is to provide a resource where individual polymer scientists can deposit their data and metadata through a robust data model. This is the idea behind MaterialsMine,^52,53^ which focuses on nanocomposite and mechanical metamaterials, and the Community Resource for Innovation in Polymer Technology (CRIPT), which considers all varieties of polymeric materials.^54^ MaterialsMine currently serves not only as a data resource, but also provides additional features on their platform to process and visualize data. For full details, we refer the reader to their Web site^52^ and article.^53^ For CRIPT, a key part of this resource will be making it easier for polymer scientists to do science through advanced search, data visualization and private data sharing prepublication. For more information, see their Web site.^54^ It builds on ideas that are already being implemented in industry,^55^ and brings them to the public domain. Furthermore, it enables polymer ML by following FAIR data practices including the use of an API (Application Programming Interface) and a web-based interface for both data deposit and access.

### Polymer Representations

For ML, chemical structures must be represented in a machine readable format. Utilizing advances in the representation of small molecules, there are a variety of methods that have been developed to address this problem. As detailed in prior reviews,^14,15^ common options include using group contribution methods, converting line notations to numerical vectors called fingerprints through open software such as RDKit,^56^ using a graph based representation along with graph convolutional neural networks to represent a 3D molecule in 2D, directly using line notation in text-based ML methods, and developing handcrafted, hierarchical fingerprints to replace or supplement the previously described fingerprints.

**Figure 2:**
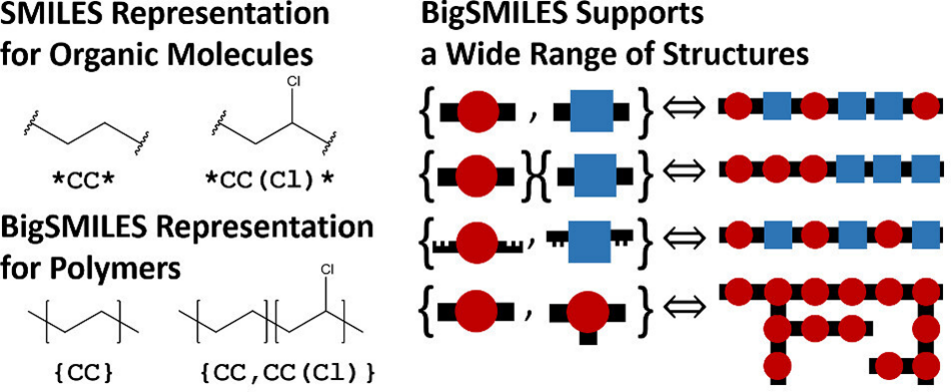
Depictions demonstrating how BigSMILES captures different polymer chemistries. Reprinted from ref (57). Copyright 2019 American Chemical Society.

Thus, far, most of these methods have focused on homopolymers ignoring the stochastic nature of polymers. A key advance in capturing the stochasticity of polymers is the development of an extension of simplified molecular-input line-entry system (SMILES) to polymers known as BigSMILES, as shown in Figure 2.^57^ More recently, PolyGrammar was developed to describe polyurethanes using a hypergraph representation.^58^ However, there is not yet a method to generate fingerprints that encode the stochasticity for all varieties of polymers. The issue of polymer stochasticity is acute for copolymers, polyolefins, and complicated polymer architectures. Kuenneth et al.^59^ find that for random copolymers, they can simply weight the homopolymer fingerprints by the relative fractions of the two monomers. However, a general solution when there are a large number of different monomers, where the ensemble plays an important role, or the structure of the polymer is nonlinear are not fully solved. One recent effort in this direction using data from simulations found that sequence defined polymers were best represented by a recurrent neural network.^60^ In a notable work, Patel et al. looked at different ways of encoding sequence and compared their results to non-sequence-specific methods.^61^ They considered four different data sets, one of which was experimental. Ultimately, they found that the best methods depend on both the property being predicted and the data set. Based on their results, they recommend encoding polymer size, including chemical based information as opposed to one hot encoding when chemistry and extrapolation are important, and making use of the polymer sequence if it is known. Recent work by Aldeghi and Coley worked to address the issue of ensembles of polymers.^62^ Specifically, they represented polymers by graphs where atoms were represented by nodes and bonds are represented by edges. Bonds between different monomers were assigned different weights based on their average probability thus allowing one to distinguish a diblock copolymer from a random copolymer. However, this work still needs to be extended to the case of conditional bonding probabilities.

Perhaps, the largest learning lesson from advances in polymer representation is that the optimal representation is highly likely to depend on the problem at hand (e.g., chemistry, ML model, task), as well as on the amount of data available for training. These interactions will often be nontrivial leading back to a key tenet of ML—that prototyping is essential. Nonetheless, basic guidance such as including metrics that matter (e.g., molecular mass if a property is sensitive to molecular mass) will continue to be important.

## New Progress

### Autonomous Experimentation

Active learning is an approach in which a ML agent, which generally consists of one or more unsupervised and/or supervised models, is responsible for choosing which data gets added to its training corpus in an iterative fashion.^32^ This approach is useful when the acquisition of the data is expensive, e.g. when the materials are costly to synthesize or the measurement is slow and tedious. A common class of active learning is Bayesian Optimization (BO) in which the property to be optimized is cast within a Bayesian statistical framework. Of particular importance in these methods is the acquisition function which determines which data point or set of data will be added to the training corpus. Common acquisition functions include pure exploration, pure exploitation, expected improvement, and Thompson sampling.^32,65^

Within the materials community, autonomous experimentation platforms are being developed to perform experiments with little to no intervention from human scientists by leveraging active learning algorithms. These automated and autonomous experiments promise to help scientists discover materials with optimized properties more quickly, map phase spaces more accurately, and use less material in the pursuit of these goals. Automated and high-throughput robotic platforms do not require breaks and can operate with higher precision and repeatability than their human counterparts. Furthermore, automated systems tend to naturally integrate with databases and materials ML platforms as the metadata for each sample is likely already digitized as part of the preparation process. Most importantly though, automated and autonomous experiments free the scientist to spend less time and energy on the tedium of running a particular experiment and more time on interpreting the data and planning the next one.

While there have been several recent studies focusing on developing active learning techniques for polymers using premeasured data sets, theory or simulations,^66−71^ here we focus on experimentally realized autonomous platforms. These studies either directly or indirectly address key challenges in applying ML to polymers as outlined in Table 1. Building automated platforms requires a confluence of skills (from machining to robotics to software development) that can be difficult to find in a single researcher or polymer research group, so these studies are often collaborative. A reality of polymer materials is they are often used or studied in nonequilibrium or kinetically trapped states and that their properties are processing history dependent. Automated platforms can help mitigate or facilitate the study of process history and nonequilibrium phenomena through their control and repeatability. Furthermore, robotic automation often provides a more direct route to quantifying certain parts of the uncertainty in material synthesis.

**Figure 3:**
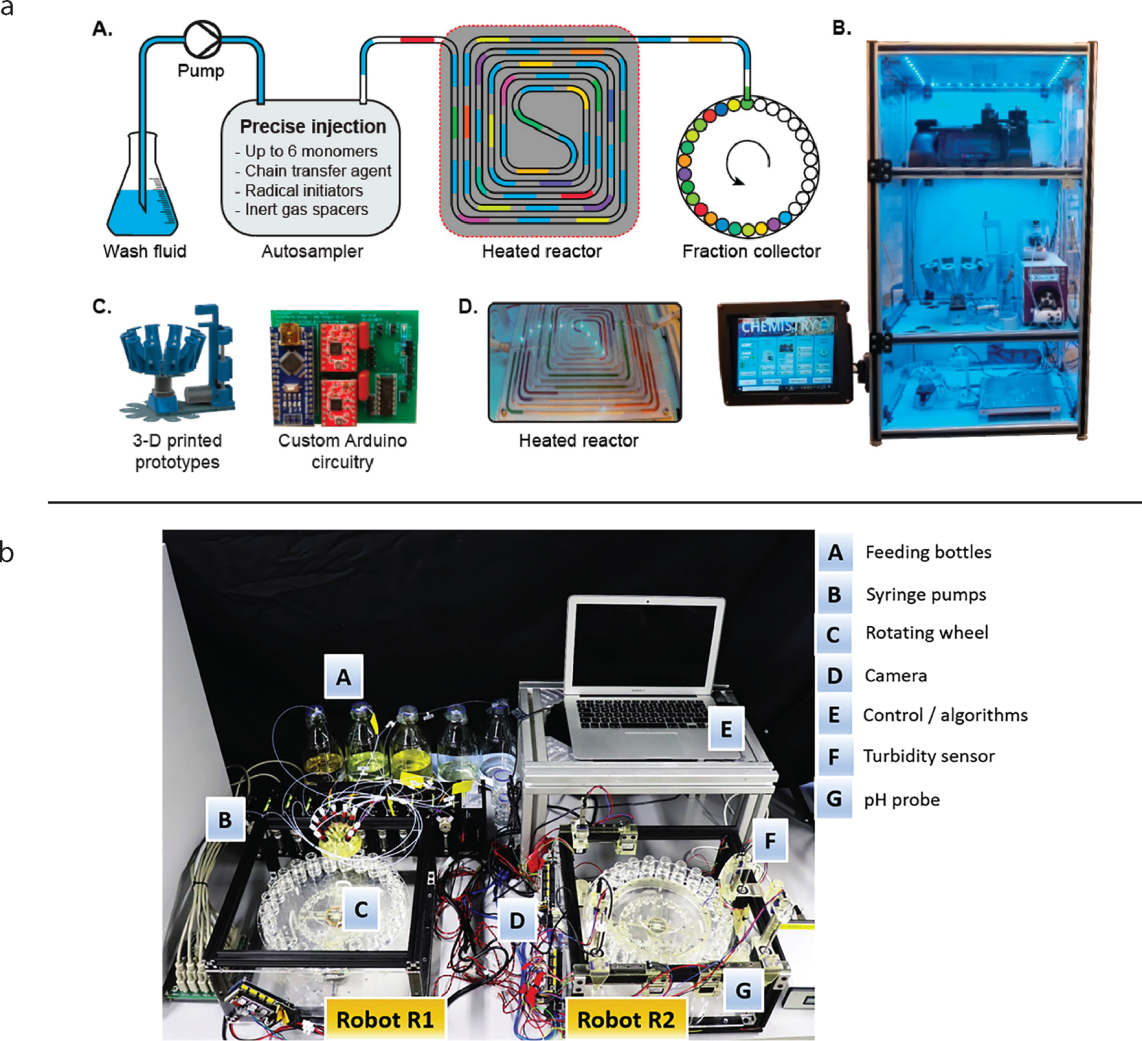
Schematics and pictures of two autonomous experimentation platforms. (a) Automated continuous flow reactor for optimizing copolymer synthesis of ^19^F MRI agents. Reprinted from ref (26). Copyright 2021 American Chemical Society. (b) Automated mixing and characterization platform for studying surfactant properties and phase behavior. Reprinted with permission under a Creative Commons CC-BY 4.0 License from ref (63). Copyright 2021 CellPress.

While significant work has gone into developing synthesis platforms and methods that mostly focus on small-molecule^72−74^ and colloidal^75−78^ synthesis, comparatively less focus has been given to polymer synthesis.^79^ In the last several years, several groups have taken advantage of the versatility of reversible addition–fragmentation chain transfer (RAFT) polymerization and constructed automated copolymer synthesis platforms.^26,80−84^ These studies seek to find the polymer sequence or reaction conditions that achieves an optimal material property such as ^19^F magnetic resonance signal (MRI) signal for contrast agents,^26^ retained enzyme efficiency for protein stabilizers,^80^ or simply the conversion and dispersity of the synthesis itself.^81^Figure 3a shows one such autonomous synthesis platform from ref (26).

Beyond synthesis, there are recent studies that focus on optimizing the design of polymer *formulations* rather than polymer *chemistry*. In these works, the goal is to find the component composition or processing conditions that optimizes some material property of interest. These include optimizing the degradation behavior of organic photovoltaic films,^85^ the gelation time and bacterial activity of living silk hydrogels,^86^ the melting point and electrical properties of deep eutectic solvents,^87^ and the physical properties and cost of surfactant solutions.^63^ The inclusion of cost as an optimization variable is notable in that it ensures that the final results balance performance against the bill of materials needed to make the sample, likely making the results more useful to industrial scientists. The idea of including secondary optimization variables can be extended to experimental nonidealities (e.g., slow motor axes, hysteresis) in order to increase the efficiency of the robotic exploration of a material property space.^88^Figure 3b shows an autonomous formulation platform from ref (63).

These studies present a mix of semiautomated^26,63,85^ and fully automated^80,81^ platforms. For semiautomated cases, the authors chose to manually perform key processing, purification, or measurement steps rather than attempting to automate them. While fully automated platforms might allow for higher throughput, the development cost, in both money and time, can often outweigh the benefit when the scientific goals of the study can be achieved with minimized, rather than zero user interaction. Furthermore, active learning researchers from outside of the polymer community point out that “human-in-the-loop” agents or “human-machine teaming” can produce better results by taking advantage of the strengths of both humans and machines.^70,89,90^

These above studies present significant variation in the kind of ML models employed in their autonomous agents. For several of the studies,^80,81^ BO approaches were used with Gaussian Process (GP) models as surrogate optimization functions. Langner et al. chose to use Bayesian neural networks in order to avoid the very poor performance scaling () that GPs exhibit with problem size.^85^ There are methods to improve the performance of GPs for large problems, but they are not necessarily applicable in all cases.^91^ Interestingly, Reis et al. avoided BO approaches entirely and instead leveraged an AutoML model which predicted ^19^F MRI signal strength from monomer composition.^26^ When using an AutoML framework, rather than choosing a specific ML model (e.g., neural network or random forest model) a variety of models are trained and automatically chosen to maximize performance.^92,93^ By evaluating this model on a grid of monomer composition, the authors could choose the compositions that the model predicted had the highest performance. While this approach loses some of the flexibility and statistical rigor of the BO approaches, it represents an simple and accessible agent to implement and embraces the prototyping nature of ML.

There are also several efforts at user facilities and national laboratories to build shareable, open platforms to enable active learning studies. The Polybot system at Argonne National Laboratory offers several stations (synthesis, characterization, processing) between which samples can be shuttled using an mobile platform with a robot arm.^94^ The Autonomous Formulation Laboratory (AFL) at the National Institute of Standards and Technology is another automation platform designed for conducting machine guided experiments on liquid formulations on neutron and X-ray scattering beamlines.^95^ These efforts are in the spirit of Open Science, which will be discussed in more detail in the eponymous section below.

### Interpretability and Explainability

Often polymer scientists desire not only the answer to a problem, such as which polymer material exhibits optimal properties, but also an understanding of why that material is optimal. In the broader ML field this is known as explainable artificial intelligence, or simply, XAI.^96^ Most efforts in XAI focus either on glass-box models, which are natively explainable (and possibly interpretable) or posthoc methods, which provide explainability for a black box model such as a neural network. This relates to the ultimate ML goal of knowledge generation.

Glass-box models, as their name suggests, provide insight into how the ML model makes predictions. This is in comparison to black-box models which only provide the prediction and no insight or explanation. Two common approaches are linear models, where the connection between input and output is straightforward, and symbolic regression, where the goal is to create an analytic function that depends on the features. One method that applies both of these approaches is the least absolute shrinkage and selection operator (LASSO) method.^97^ The basic concept behind LASSO is to combine linear regression with a regularization term that encourages the learned prefactors to be exactly zero, as opposed to small values as in Kernel Ridge Regression.^98^ The regularization is controlled through a prefactor with larger values corresponding to fewer nonzero prefactors in the linear regression. Thus, LASSO can be used to create linear models that are intrinsically interpretable. It can also be used for symbolic regression by creating a large number of potential terms by combining features through simple or complicated functions (e.g., *x*_1_*x*_3_^2^ where *x*_1_ and *x*_3_ are features) and then selecting only the most salient terms. Two limitations of LASSO are its inability to handle both very large numbers of potential terms and highly correlated terms. To overcome these challenges the sure independence screening and sparsifying operator (SISSO) method was developed.^99^ SISSO first creates a very large () number of features. Then sure independence screening (SIS) is used to correlate the features with the target output keeping only the highest ranked features. Next, a sparsifying operator (SO) is applied to determine the optimal n-dimensional feature vector. This process continues for successively larger n-dimensions until a target error is achieved. Pilania et al. used SISSO in two different ways.^100^ In the first case, they approached the problem via interpretability by selecting the single most important feature that is a function of the original selection of features. This resulted in an analytic model for the glass transition temperature of polyhydroxyalkanoate polymers with excellent error. In the second case, they used SISSO to create enhanced features under the assumption that mathematical combinations of features that are better correlated with the target property should improve performance compared to using the original features directly.

**Figure 4:**
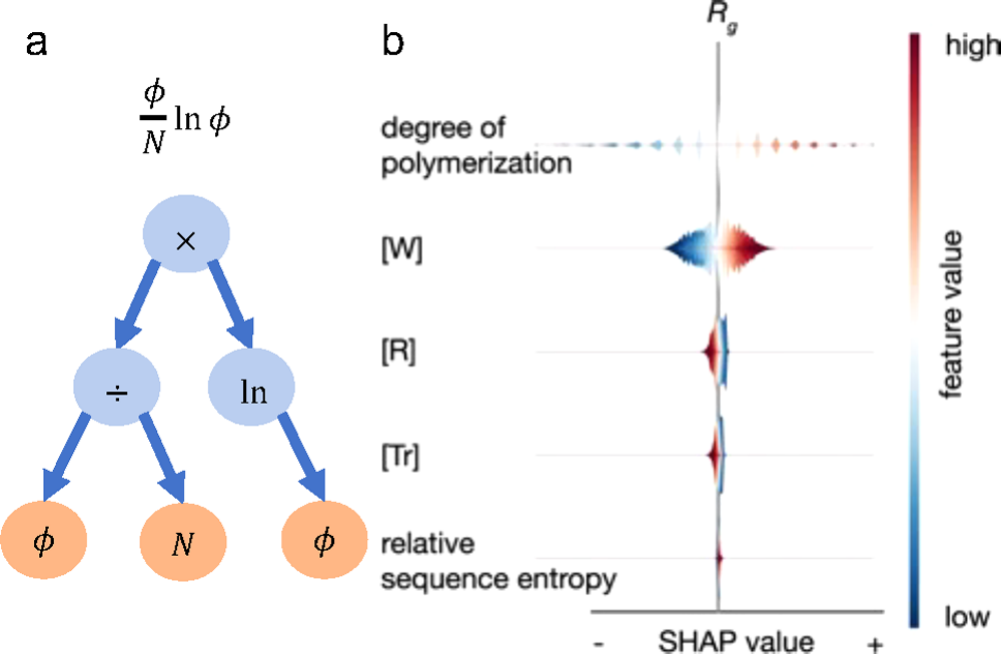
(a) Tree representation of the equation for polymer entropy (ϕ/*N* ln ϕ). Features (ϕ and *N*; depicted in orange) are leaves and operators (e.g., ×; depicted in blue) are nonterminal nodes. (b) SHAP values for the prediction of the radius of gyration (*R*_g_) of sequence defined copolymers. Features including degree of polymerization, monomer in a good solvent ([W]), monomer in a bad solvent ([R]), monomer in a theta solvent ([Tr]), and relative sequence entropy. The figure shows that the degree of polymerization has the largest effect of the features and that larger degrees of polymerization correspond to larger values of *R*_g_. Monomers in a good solvent have a similar interpretation. Monomers in bad solvent and theta solvent are anticorrelated and have a significantly smaller effect on *R*_g_. Reprinted in part with permission under a Creative Commons CC-BY 4.0 License from ref (34). Copyright 2021 The Authors.

Symbolic regression can also be implemented in other ways. For example, in genetic programming symbolic regression (GPSR)^101^ a different approach is taken to yield an analytic expression that describes the output as a function of a subset of the features. Here, both a list of features and a list of mathematical operators (e.g., *+*, *–*, ×, ÷) are provided. They are then represented as a tree with the features as the leaves and operators as nonterminal nodes as depicted in Figure 4a for the expression for polymer entropy. The optimal tree is then determined using evolutionary algorithms such as a genetic algorithm. The benefit of this method is that the search is potentially performed over a larger space. Although, GPSR has yet to be applied in the polymers domain to our knowledge, it has been used for other materials.^102,103^

There are also other techniques such as explainable boosting machines (EBMs).^104^ EBMs are a form of a generalized additive model where the output is typically a sum of nonlinear and nonsmooth functions of each feature. This means that the relative contribution of each feature on the output is trivial to discern. Although they can be slow to train, evaluation is quick and accuracy can be on par with black-box models. Instead, the main limitation is that the additive model assumption may not be an accurate assumption for every system or problem.

There are also a variety of posthoc analysis methods. The most common methods are SHapley Additive exPlanations (SHAP)^105^ and Local Interpretable Model-Agnostic Explanations (LIME).^106^ Both of these approaches are model agnostic. SHAP takes a game theoretic approach to determine the impact of all the features on a given output. Specifically, for a single training data point, each feature is assigned a SHAP value where the sum of all of the SHAP values is equal to the difference between the given output and the expected output across all of the training data. These SHAP values can then be computed across the entire training data set to give an overall understanding of how different features affect the predicted results including the magnitude of such predictions. An example of such a plot is shown in Figure 4b. LIME uses a different approach. First, one chooses a particular output that they want to explain. Then the input is perturbed in various ways. Next, a local (often linear) model is trained weighting data points that are closer to the desired state that was queried. The local model can then be used to describe why the original ML model made its predictions for a given instance.

In the context of polymers, SHAP has been used to investigate the contributions of various features.^34,80,107,108^ For example, it has been used to determine which functional groups and polymer properties are most predictive of membrane permeability.^107^ It has also been used to look at the effect of monomer type and degree of polymerization on protein stability for polymer–protein hybrids. In this example, they also used active learning and probed how the SHAP values changed as a function of the iteration.^80^ Recently, Amamoto et al. used both SHAP and LIME to understand important regions in 2D wide-angle X-ray diffraction and small-angle X-ray scatting measurements when using convolutional neural networks to predict polymer type and annealing temperature.^109^ Although more simplistic than both SHAP and LIME, partial dependence plots (PDP), which show the marginal effect of only one or two key features can provide qualitative guidance. For example, Bejagam et al. look at the two most important features and determine its nonlinear effect on the melting temperature.^110^ Ultimately, they conclude that molecular compactness plays a key role.

### Data Fusion and Transfer Learning

The goal of data fusion is to achieve synergy by combining, potentially several, but at least two different data sets. This is analogous to the motivation for multimodal measurements in a non-ML context.^111^ Data fusion can be accomplished both in the context of supervised and unsupervised learning. However, as detailed in a general review,^112^ there are still a variety of outstanding issues such as combining different data types and accurately handling uncertainty.

**Figure 5:**
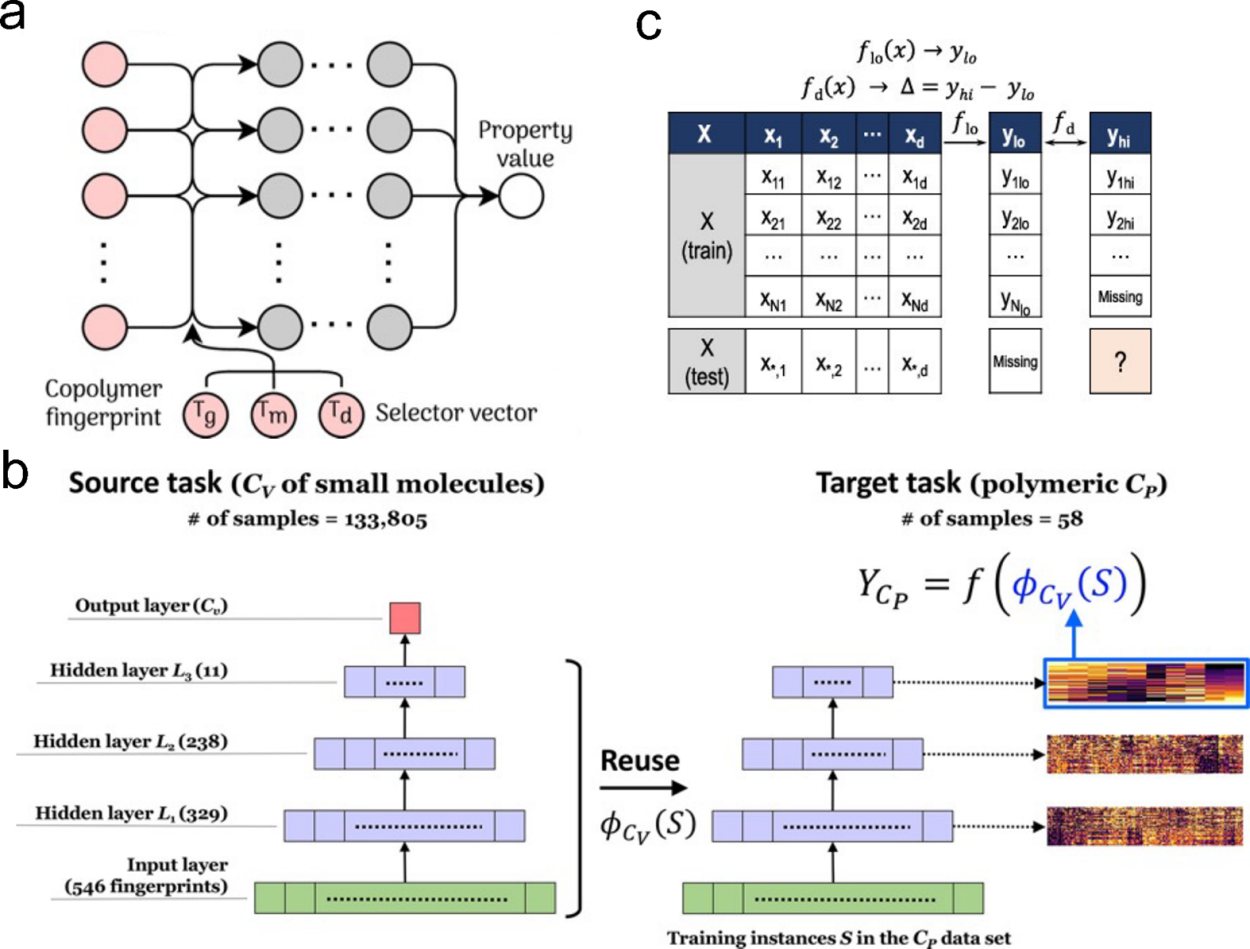
(a) Example of multitask learning, where the property of interest is fed in as an additional one hot encoded vector. Properties to predict include the glass transition temperature (*T*_g_), the melting temperature (*T*_m_), and the degradation temperature (*T*_d_). Reprinted from ref (27). Copyright 2019 American Chemical Society. (b) Example of reusing nodes from a neural network trained on small-molecule-specific heat capacity at constant volume (*C*_*v*_) to predict polymeric-specific heat capacity at constant pressure (*C*_p_). Reprinted in part from ref (59). Copyright 2021 American Chemical Society. (c) Example of multifidelity modeling. Reprinted from ref (115). Copyright 2020 American Chemical Society.

Nonetheless, data fusion has already shown promise in polymer science, specifically in the form of multitask learning where one model is used to predict multiple quantities. Kuenneth et al. have shown that it can be used to simultaneously predict 36 different polymer properties.^108^ Ultimately, they found that multitask learning where the desired property is encoded via augmenting the feature input with a property selector works better than either having the ML model predict all of the properties as an output, or predicting each property individually. This is directly a consequence of using neural nets as their ML model and, during model training, this mode of operation allows the optimizer to more effectively use sparse data. A graphical depiction of this scheme as applied to random copolymers^59^ is shown in Figure 5a. Multitask learning has also been used to predict properties from images of nanocomposites using a convolutional neural network^113^ and to simultaneously denoise and predict sample characteristics from X-ray hyperspectral images using an autoencoder.^114^

Related to data fusion, transfer learning is a ML technique where information is transferred between tasks (e.g., predictions of glass transition temperature or melting temperature), domains (e.g., polymer literature or webpages) or both. Since information is often transferred from a data-rich task or domain, known as the source, to a data-poor task or domain, known as the target, it allows for improved predictions for the target for smaller data set sizes. Challenges with transfer learning include selecting the appropriate source, selecting the optimal ML model and, critically, how the information is transferred. We refer the reader to an excellent general review on the topic.^116^ Within polymer science, transfer learning is increasingly being used.^27,117−121^ For example, Li et al. used it to reconstruct microstructures and generate structure–property predictions for nanocomposites. In this particular case, they use a deep convolutional neural net trained on a nonscientific corpus for their source domain.^117^ Transfer learning has also been used to make property predictions for extremely small data sets by Yamada et al. Their approach involved generating a large number of potential models that predicted other properties. These models varied both in the model itself, as well as the target property. One example using neural networks is shown in Figure 5b. They then tested all of the models and determined which ones performed best. This allowed for accurate predictions with data set sizes of .^27^ Most recently, Lu et al. first trained an unsupervised encoder on TEM images. Then they transferred this encoder to perform other tasks such as morphology classification and nanowire segmentation. Ultimately, for morphology classification, they found they needed less than 10 labeled images per class and, if the underlying distribution was known a priori, only a single labeled image per class was necessary.^121^ These results are particularly exciting, since manual labeling of data is time-consuming and error prone.

Another related concept is that of multifidelity models. These models can be thought of as transfer learning where the source task and the target task predict the same quantity, but at two different levels of fidelity, or accuracy. In this case, the source task is the lower fidelity model that is data-rich, while the target task is a higher fidelity model that is data-poor. For this particular case, a common approach is to train the high fidelity model to learn the scaled difference between the data and the low fidelity model.^122^ As an example, Venkatram et al. used this technique to predict the tendency to crystallize as a function of chemistry with the high fidelity data set composed of experimental results and the low fidelity data set composed of predictions from group contribution methods.^115^ This scheme is shown in Figure 5c. By making use of the low fidelity information, they were able to reduce their root mean squared error by almost a factor of 2 compared to a model trained on only the high fidelity data. Similarly, this approach has also been used to predict polymer bandgap.^123^

### Domain Knowledge

A promising area that is just starting to gain traction in ML as applied to polymer science is the idea of using domain knowledge—our cumulative knowledge of polymer science—to enhance ML models. Although this is not a new idea in ML it is a powerful one, as detailed in two general^124,125^ and one materials focused^126^ reviews. It is also related to the concept of inductive bias,^127^ where models are modified to bias toward certain solutions over others independent of the training data (e.g., enforcing known constraints). Ultimately, incorporation of domain knowledge can potentially improve both interpolation and extrapolation for the small data set sizes that are common in polymer science. Furthermore, in principle, domain knowledge can be leveraged to address process history dependent data.

Incorporating domain knowledge can range from conceptually simple to complex. Domain knowledge has commonly been used to select the appropriate features^128^ for a ML model or to enforce known constraints^119,129^ such as transitional invariance. In both of these cases, less data is needed for the same accuracy for interpolation and, in many cases, extrapolation as the constraints and feature correllations do not need to be learned.

**Figure 6:**
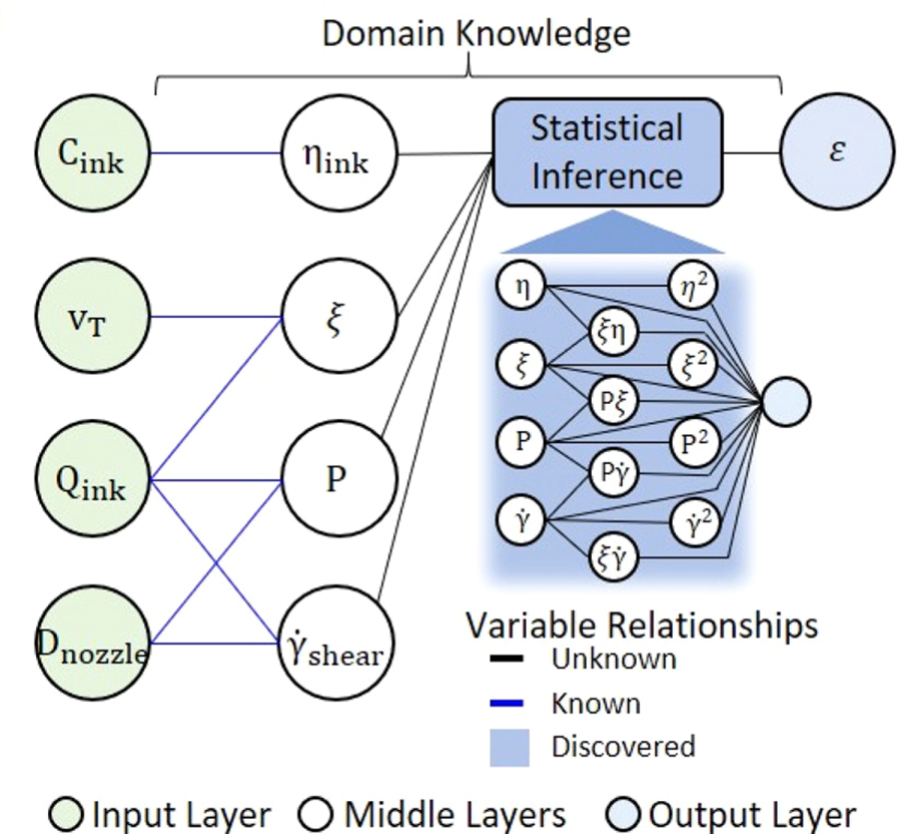
Example of hierarchical ML as applied to 3D printed biopolymers. The input, including polymer concentration (*C*_ink_), nozzle speed (*v*_T_), flow rate (*Q*), and nozzle diameter (*D*_nozzle_), is linked to the middle layers represented by physical quantities such as ink viscosity η_ink_ through simple physical models. Statistical inference in the form of LASSO then used to predict the desired quantity of the difference between the expected and the observed dimensions (ϵ). Note that the feature space for LASSO was extended by considering second order terms. Reprinted in part from ref (25). Copyright 2020 American Chemical Society.

An exciting idea for incorporating domain knowledge is to make use of theory, which has the possibility of not only improving the ML models, but also working toward explainability and interpretability. For example, Menon et al. developed a hierarchical ML approach.^130^ First, they use simple physical models to predict basic physical properties. Then, they use the physical properties as features for LASSO to predict a complicated target property. The physics are directly included via the simple physical models, and the final expression is explainable due to the use of LASSO as depicted in Figure 6. More recently, Audus et al. explored different methods for incorporating imperfect theory into ML models with the goal of improving interpolation, extrapolation and explainability.^131^ Using the simple case study of the size of a single chain in different solvent qualities, they found that as one incorporates more knowledge all of the key metrics improved. They also found that, when the numerical values of the theory were encoded, predicting the difference between the theory and the data performed best, but that further improvement could be achieved by using the functional form of the theory. Incorporating the full functional form of the theory had the added benefit of being easy to interpret.

### Deep Learning

**Figure 7:**
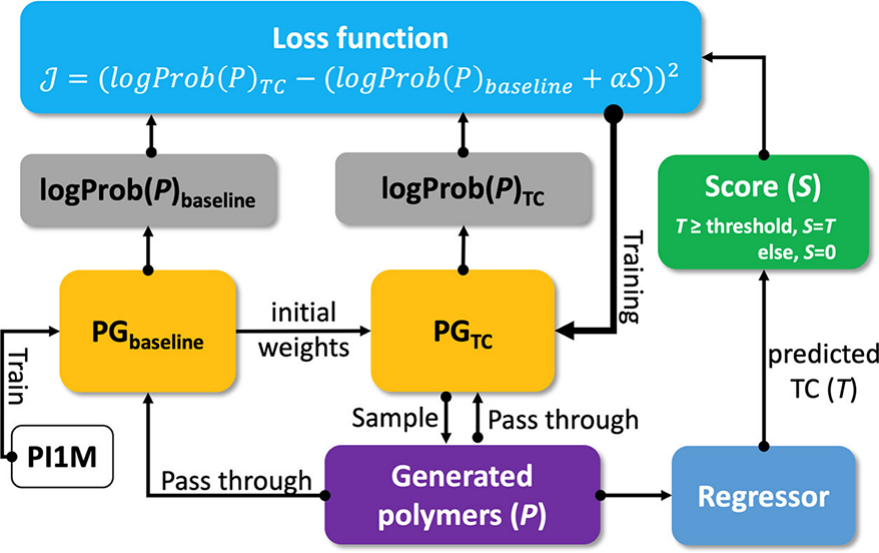
Reinforcement learning scheme to generate polymers with high thermal conductivity (TC). PG_baseline_ is a polymer generator trained on the PI1M data set. PG_TC_ is a polymer generator that is trained to maximize TC. PG_TC_ is sampled to create the generated polymers (P), which are passed to the regressor to predict TC. The generated polymers and TC are the used to calculate the loss function used in training PG_TC_. Reprinted from ref (33). Copyright 2022 American Chemical Society.

Another trend in ML in polymer science is the use of advanced deep learning techniques. Examples include recurrent neural networks (RNNs), which are designed to handle sequences such as the sequence in a copolymer,^21,23,60,61,120^ variational autoencoders (VAEs), which are composed of an encoder and a decoder with a smaller latent space in between,^114,132^ reinforcement learning (RL),^33^ where an agent takes an action and then receives a reward, generative adversarial networks (GAN),^133^ composed of a generator and a discriminator, and graph neural networks (GNNs),^62^ which are designed to handle graph based data such as a polymer structure. An example of RL is shown in Figure 7. For a detailed description of these methods, we refer the reader to recent reviews.^7,8,14,15^

### Enhanced Scattering

In small-angle X-ray and neutron scattering (SAS) of polymer and soft material systems, the challenge of interpreting data often matches or exceeds the challenge of preparing samples or conducting the experiment itself. This is in part due to the nature of the measurement and in part due to the great variety of microstructures that polymer materials exhibit. In order to interpret SAS data, there exists a library of geometric and phenomenological analytical models that researchers much choose from in order to extract physical meaning from the measurement.^135^ Due to the “phase problem”,^136^ there are likely many models that will fit a measured data set (as described by χ^2^ minimization), even if they are not proper descriptors of the underlying structure. This makes choosing the correct scattering model a difficult but incredibly important task.

**Figure 8:**
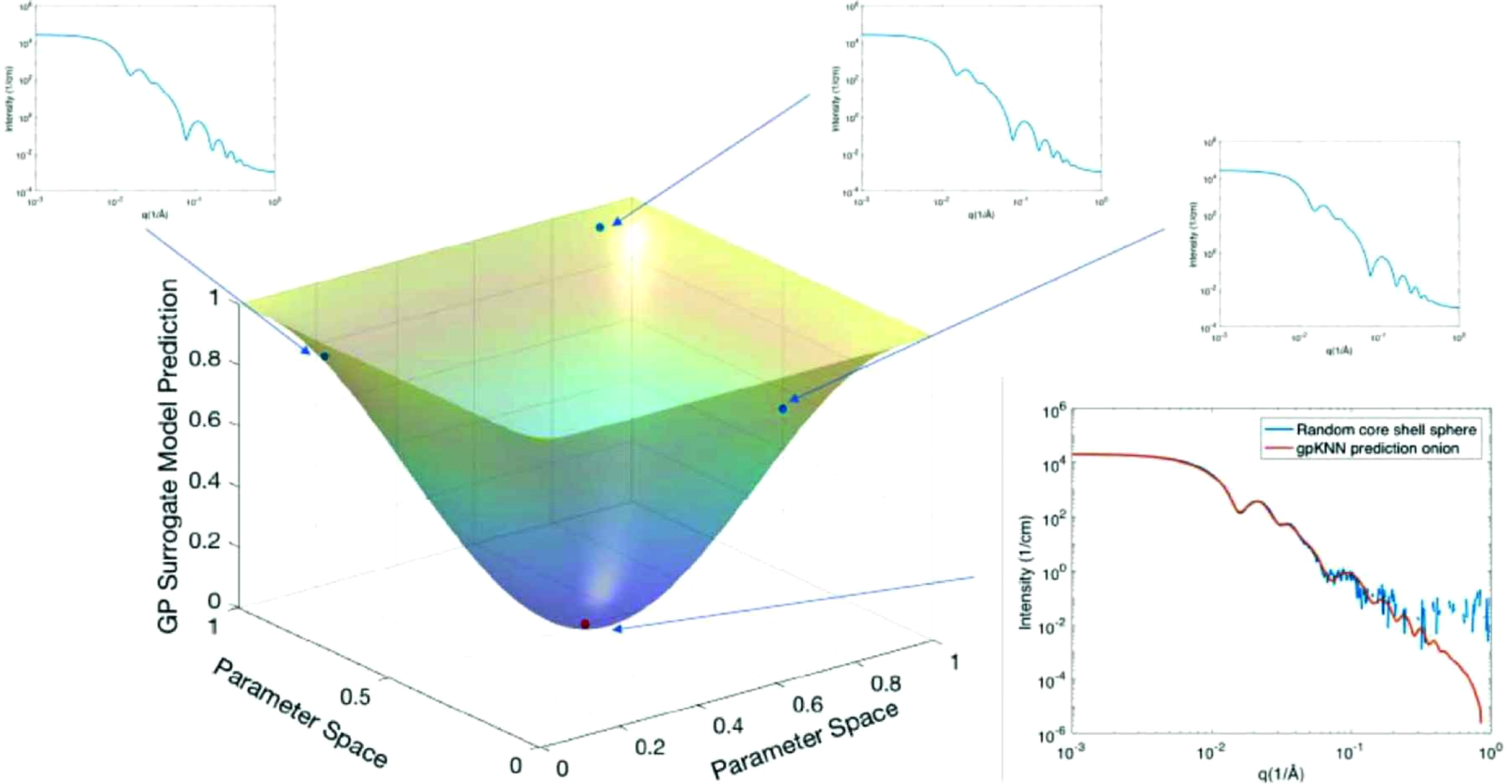
Result of an ML-guided fitting process for small-angle scattering data. The neighborhood of the closest scattering model found is used to generate interpolations to determine whether a better fit to the data can be found. Reprinted with permission from ref (134). Copyright 2020 International Union of Crystallography.

In light of this, several authors have developed ML models which attempt to guide the users toward the most probable analytical models that describe their data.^134,137,138^ In all cases, the authors constructed a library of theoretical data and explored a variety of supervised ML algorithms (including AutoML). Figure 8 shows the goodness-of-fit surface calculated using a GP that is interpolating across the parameter space of a complex scattering model. By combining this surrogate model with a k-Nearest Neighbors classifier, the authors are able to identify the correct scattering model for a given data set with high accuracy.^134^ They show that using the GP as a surrogate model, rather than using the analytical models directly, considerably increased the number of times that the correct model appeared in the top three predictions of the classifier. While similar to this work, the software package from Politi et al. is also notable in that it includes automated feature engineering in order to increase the classification accuracy of the overall method.^138^

In addition to model-selection schemes, Jayaraman and co-workers have developed the computational reverse-engineering analysis for scattering experiments (CREASE) method which seeks to reconstruct three-dimensional structures of polymer materials from SAS data using genetic optimization.^139−141^ These authors leverage supervised, surrogate models in lieu of expensive analytical and simulation computations in order greatly reduce the convergence time of their method.

### Unsupervised Analysis

Dimensionality reduction is a class of unsupervised approaches for analyzing unlabeled data. These methods can validate old wisdom and provide new insight into data sets because they rely on fewer assumptions and a priori knowledge (i.e., labels) than many supervised methods. For example, these approaches were recently used to reconstruct the periodic table from just a feature vector composed of simple atomic properties.^142^ In addition, unsupervised methods provide a path toward leveraging data that is too large, tedious or complex to analyze or label by hand e.g., from high-throughput experiments or large scale simulations. The trade-off for dimensionality reduction methods is that interpreting the meaning of data projected onto an unknown subspace can be challenging. Despite this, recent works have leveraged these methods to positive effect.

Several groups have used unsupervised methods to analyze the local and global 3-D structure of polymer simulations.^143−145^ Parker et al. surveyed various unsupervised (and supervised) methods for the task of identifying conformational transitions of polymers adsorbed to nanowires.^145^ A key finding of this work is that, while all unsupervised methods surveyed were able to distinguish the different conformations of the polymer, most required specific data prepossessing to be effective. Statt et al. used the Uniform Manifold Approximation and Projection (UMAP) method to understand copolymer assembly as a function of monomer sequence.^143^ UMAP is a nonlinear manifold learning technique that focuses on preserving both the local and global structure of data.^146^ Using UMAP, the authors were able to not only identify common global structures in their simulations (e.g., strings, membranes, vesicles) but also how much each monomer contributed to a structure. These results were exemplified in 3-D simulation snapshots where the beads were colored by “structure”, a unique and powerful way to analyze heterogeneous simulation structures.

**Figure 9:**
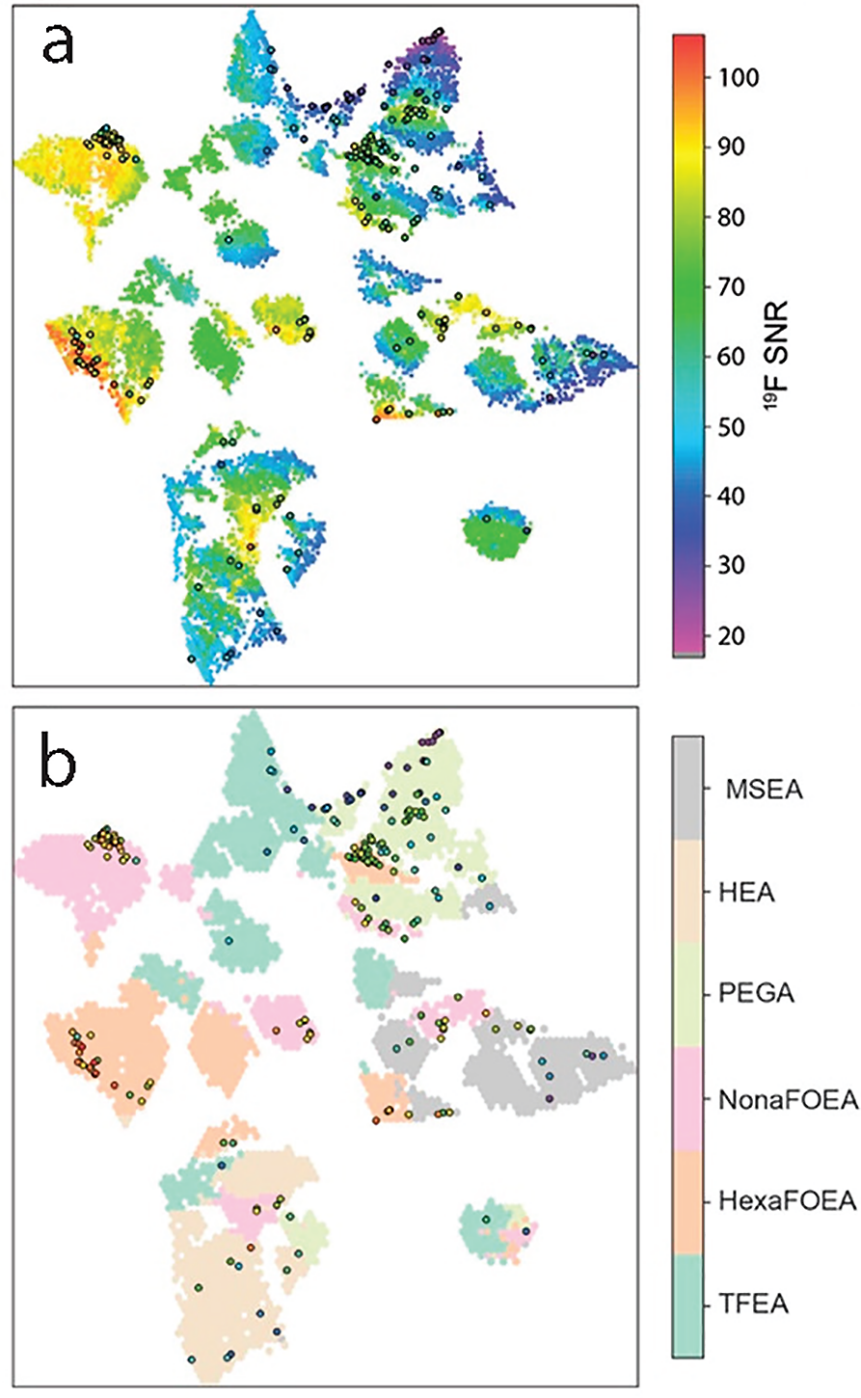
(a) UMAP projection of a copolymer compositional space with the ML predicted ^19^F MRI signal-to-noise color coded. Circled samples represent experimentally validated water-soluble structures. (b) UMAP projection of a copolymer compositional space with the major comonomer component color coded. Circled samples represent experimentally validated water-soluble structures. Reprinted in part from ref (26). Copyright 2021 American Chemical Society.

Researchers have also used UMAP to better understand the chemical origins of optimized materials found via active learning algorithms.^26,34^Figure 9 shows a UMAP projection of a copolymer computational space that was explored using an autonomous platform while trying to optimize ^19^F MRI signal.^26^ These data show that areas of highest signal fall into chemically similar regions and that two of the six comonomers dominate the high-signal regions. While this observation could likely have been learned by careful analysis of the data itself, the UMAP projection makes the conclusion clear and obvious.

Somewhat analogously to the above autonomous studies, Rodriguez et al. used Principle Component Analysis (PCA) and T-distributed stochastic neighbor embedding (t-SNE) as visualization and screening tools.^87^ They leveraged these methods to visualize and aid in the process of down-selecting candidates for high-throughput analysis from a material library that was too large to analyze in full.

### Optimization and Inverse Design

As previously stated, one of the ultimate goals of ML for polymers is materials/process design. This often takes the form of optimization, most commonly property optimization.^22−24,63,110,118^ It is important to note that while most efforts have focused on optimizing the chemistry or formulation, one can also optimize the materials processing steps (e.g., annealing, film casting, mixing conditions). One can also simultaneously optimize multiple quantities.^34,63^ Materials optimization falls into the category of inverse design where the goal is to find an input (e.g., synthesis or processing parameters) that yields a desired output (e.g., material property). Inverse design can include other components such as generative models and high-throughput screening.^33,132^ For example, Ma and co-workers use a polymer generator that maximizes the thermal conductivity (see Figure 7).^33^ For additional details on inverse design, we direct the reader to a recent, comprehensive review by Sattari and co-workers.^15^

## Outlook

### Interpretability and Explainability

As ML continues to mature for polymer science, we expect to see an increase in the focus on interpretability and explainability. A better understanding of the ML model means that the user will have an improved intuition on when the model may extrapolate accurately or when it might fail which should accelerate the discovery of new knowledge.

However, the appropriate use of improvements in interpretability and explainability will depend on the specific problem and data availability. For example, linear models and their extensions, including generalized additive models or use of basis functions, provide clear connections between inputs and outputs. However, the underlying assumptions of these models may not be valid. For example, EBMs will be unable to correctly capture a complicated nonlinear relationship between two features. Thus, they must be used carefully, recognizing that while the models are explainable, they may not represent the true underlying physics. Symbolic regression will be particularly powerful when applied to problems where an analytic solution exists but is unknown. Since this assumption may not be valid, one potential path forward is to break up the problem into different regimes each with its own analytic solution. Symbolic regression can then be used separately in those different regimes to get different expressions depending on the features. To determine such regimes, one can use unsupervised clustering techniques first. In the future, it will be interesting if symbolic regression can be extended to consider more complicated operators such as integrals and derivatives, which will extend the power of symbolic regression, although at the expense of additional complexity.

Since the aforementioned glass-box models ultimately have their limitations, there will still be a place for complex, black-box ML models such as deep neural networks or graph neural networks. In these cases, we expect to see increased use of posthoc explainability techniques such as SHAP and LIME. Although these techniques focus on local rather than global explainability, they can still provide knowledge in addition to the predictions from the models.

### Data Fusion and Transfer learning

Whether data fusion is successful or not will ultimately depend on the context. Data fusion will be the most successful when desired quantities are correlated, allowing knowledge of feature representation for one task to be related to feature representation for another task. This can partially be determined in advance by explicitly looking at correlations between desired quantities. Ultimately, methods such as multitask learning may benefit some predictions but not others. Nonetheless, data fusion is potentially a powerful way for imputing unknown values in scarce data sets that are common in polymer physics.

As general advances are made in transfer learning, they can often be adapted to the polymers space. In the future, it will be interesting to see what the full toolbox of techniques look like beyond the current commonly used methods such as freezing parts of neural networks, learning the difference between the source and the target, and augmenting the target with the source. Even as the toolbox is built out, the role of prototyping will still likely be important as demonstrated by Yamada et al.^27^

As such advances in transfer learning continue, they are also likely to impact multifidelity models, since a multifidelity model can be thought of as a transfer learning problem where the low fidelity, data rich task serves as the source while the high fidelity, data poor task serves as the target. However, advances in multifidelity are not necessarily limited to transfer learning. Instead the multifidelity nature can be explicitly taken into account by utilizing the relative cost of generating low fidelity data versus high fidelity data, e.g., in the context of active learning.

### Domain Knowledge

The decision of when to apply domain knowledge can be thought of by considering first where one is on the spectrum of knowledge and data. At one extreme, we have perfect understanding of a system; in this case, data is not required. At the other extreme, there is data but a complete lack of knowledge. Almost all cases fall in between. As long as polymer science and ML continue to reside in a data poor and domain knowledge rich regime, we expect the use of domain knowledge in ML to grow by leveraging advances in related fields such as transfer learning and explainability. Important considerations when choosing to incorporate domain knowledge include whether soft or hard constraints should be used, how best to capture a polymer scientist’s intuition, how much data is available, if extrapolation is important and, finally, the role of explainability.

The choice of the type of constraint can be very important; some problems involve hard constraints such as translational invariance in simulations whereas others such as phase equilibria may seem to have such constraints but in practice do not due to kinetics. In the latter situation, soft constraints nudging the system in the correct direction but allowing violation of the constraint are critical to avoiding overconfidence and negative transfer in a transfer learning context. There is also the issue of how best to incorporate domain knowledge, which is a developing field. This can range from the examples already provided to having polymer scientists create training data for an ML model, for example, by encoding their intuition via providing a probability of a material being of use. This can be further advanced by leveraging ideas such as active learning. The amount of data is also important. In more complex, data rich environments, it may be more fruitful to use ML to learn the best features rather than rely on intuition. For example, Wang et al. showed that, for the task of structure property prediction, using a convolutional neural network works better than the traditional, intuitive two point statistics as input for a neural network.^113^ There is also the consideration of extrapolation. Using domain knowledge can potentially prevent unphysical extrapolation. Finally, in some cases incorporating domain knowledge can be used for explainability. One interesting approach is to use ML to learn a simplified representation. For example, Cubuk et al. use ML to generate a structural quantity that predicts microscopic rearrangements and show that it correlates strongly to measures of plasticity in glassy systems.^147^

When these considerations are taken into account, incorporating domain knowledge in ML has a exciting future as ML can enhance qualitative data, such as from coarse-grained simulations or theories, and potentially even elevate it to be quantitative. In this context, ideas from multifidelity modeling will also be relevant.

### Open Science

**Figure 10:**
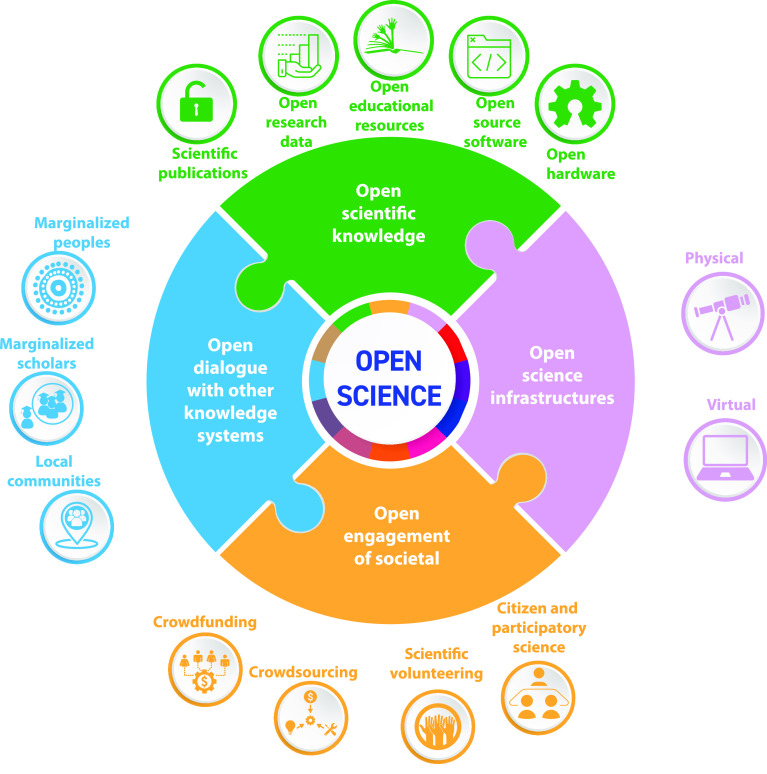
Schematic of the UNESCO defined pillars of open science. Adapted with permission under a Creative Commons CC-BY-SA 3.0 IGO License from UNESCO Recommendation on Open Science; https://unesdoc.unesco.org/ark:/48223/pf0000379949 (accessed 2022-11-26).^148^ Copyright 2021 UNESCO.

Increasingly, there is a community led effort toward adopting the principles of Open Science. While different authors define Open Science differently, a recent comprehensive review defines Open Science as “*the transparent and accessible knowledge that is shared and developed through collaborative networks.*”^150^ A United Nations Educational, Scientific, and Culturual Organization (UNESCO) workshop report states that “*the core values Open Science stem from the rights-based, ethical, epistemological, economic, legal, political, social, multi-stakeholder and technological implications of opening science to society and broadening the principles of openness to the whole cycle of scientific research*”.^148^Figure 10 highlights these core values. Working toward these goals can be achieved via open source journals such as *ACS Polymers Au*, use of preprint servers such as arXiv^151^ and ChemRxiv,^152^ sharing of data or the sharing of code. There are also efforts such as the MLExchange that seek to provide an easy interface for users to store, share, and execute their ML models.^153^ It has previously been found that in general, the benefits to individual researchers for following Open Science principles are numerous including increased citations and funding opportunities.^154^

There are at least four large barriers that often prohibit scientists from broadly sharing data: understanding best practices, knowing where to put the data, knowing how to represent the data, and having the time to clean and, ultimately, share the data. The currently accepted best practice and gold standard is FAIR data. The principles behind FAIR data are explicitly detailed in Box 2 of Wilkinson et al.^38^ and provide a simple checklist for a researcher to determine if their data is FAIR or not. However, some items in the checklist still need to be addressed by the larger community. For example, “R1.3. (meta)data meet domain-relevant community standards” supposes that a community standard exists. Such topics are currently being addressed by the Materials Research Data Alliance (MaRDA).^155^ For where to put the data, there are several options available such as the Materials Data Facility,^156,157^ Zenodo,^158^ figshare,^159^ MaterialsMine,^53,160−162^ a Community Resource for Innovation in Polymer Technology (CRIPT),^54^ institution-specific resources, *etc*. To help make data FAIR, *Scientific Data* serves as a peer-reviewed, open-access journal for describing research data sets, which has already been used for polymers (e.g., ref (40)). In terms of how to represent the data, this is still an area of active research being addressed by MaRDA,^155^ MaterialsMine,^53,160−162^ CRIPT,^54,163^ and others.^164^ However, the single largest barrier to FAIR data is the time that is necessary to clean and share the data. Thus, the efforts of MaterialsMine and CRIPT are notable as they provide additional benefits with the goal of making things net easier for polymer scientists to deposit their data. For example, MaterialsMine provides advanced visualization and CRIPT provides advanced search capabilities allowing one to find similar chemistries, polymer architectures and properties, among others.

Combined with the advances in shared data, it could be argued that the explosion of ML research in materials science was partly catalyzed by the existence of open source ML codebases.^165−170^ While this is a code-centric perspective, the ability of a nonexpert to sample various powerful ML algorithms cannot be minimized. A group whose expertise is in polymer synthesis does not need to learn advanced graphics processing unit (GPU) programming to train a convolutional neural net, they simply install Tensorflow or Pytorch and download a network model from Github.^166,167^ A formulation engineer can leverage Bayesian optimization and GP calculations without a formal background in statistical modeling.^168−171^

While the importance of open software for materials science is not a new idea,^172^ the broad application of ML techniques by nonexperts has brought about new challenges. While open packages make it easy for everyone to leverage powerful techniques, they do not always force users to use them *correctly*. It is important that developers include heavy guardrails, error checking, and documentation in their codebases to ensure that their tools are used correctly. Furthermore, it is imperative that, upon publication of their work, researchers provide their analyses and ML codebases for other groups to use and scrutinize. When code is released, is important that the code is written with good software engineering principles in mind so that it can be easily maintained and used by the community. These principles include concepts like version control, automated unit testing, code style guidelines, user and API documentation and semantic versioning. Organizations like Software Carpentry^36,173^ seek to increase the code literacy among scientists, but, as of the writing of this article, do not include detailed software engineering principles in their educational materials beyond covering version-control. The importance of code-sharing, software engineering principles, and detailed documentation need to be emphasized in research funding and by publishers and principle investigators.

### Best Practices and Challenge Problems

Similar to all specializations within polymer science, ML also has its own set of best practices to ensure good science. A recent guide focused on materials in general^174^ details several of these. This guide breaks best practices into categories such as data, modeling, benchmarking, and reproducibility. For example, data best practices include choosing a data set, data set composition, use of uncertainities and splitting train-validation-test splits, while modeling includes model choice, data scaling, and hyperparameter optimization. Prototyping is particularly important in ML. There are many times, where it is not a priori clear which models or which data manipulation techniques, such as normalization, will work best. Ultimately, the shortest route to production code will be trying different things and finding which works best. In the context of polymer design, a key test of the model is if it can be used in production, which may require the synthesis of new molecules.^22,118^ While this may be a tedious or costly step, it is important if model is to be truly validated. Reducing the barrier to experimental validation is part of the motivation for the development of automatic and autonomous experimentation platforms.

Related to this, benchmarking is particularly important as benchmarking allows other researchers to understand when various models work better than others and if there are any general conclusions. For example, as detailed in Polymer Representations, benchmarking has clarified that, to date, there is no single best representation that works for all problems.^175^ Benchmarking is not only limited to feature selection, but also includes model selection and data selection.^45,176^

From a broader community perspective and related to benchmarking is the idea of grand challenges, such as the critical assessment of protein structure prediction (CASP) competition for protein folding,^177^ which accelerates progress within subfields on important problems. Specifically, the need for grand challenges has been called out in the Materials Genome Initiative Strategic Plan released in November of 2021.^178^

### Beyond Polymer Autonomous

While the polymers community has started to develop and adopt autonomous and high-throughput techniques, the greater (hard) materials and chemistry communities have been rigorously pursuing this field as evidenced by the many recent reviews.^72,74−77,90,179,180^ While there are some barriers to directly adopting techniques from these fields, there is still much to learn and adapt from them and we will discuss a few key results here.

There have been many nonpolymer studies on the topics of autonomous formulation exploration and phase-mapping and many of them make use of theory informed or constrained models, similar to what was discussed in the Domain Knowledge section above.^88,181−188^ In order to increase the accuracy of their phase-identification from X-ray diffraction measurements (XRD), Suram et al. used a customized non-negative matrix factorization (NMF) approach in which they incorporated physical knowledge of solid state phase diagrams such as Gibb’s phase rule and XRD peak-shifting due to alloying.^184,185^ Under similar motivations, Chen et al. used an unsupervised, autoencoder approach in which they construct a latent subspace of meaningful variables and then express constraints with these variables.^182^ Kusne et al. also leveraged domain knowledge in their agent but, interestingly, also demonstrated that employing multitask learning to combine the task of property optimization with that of identifying phase boundaries is more efficient than performing either task alone.^181,187^ Finally, McDannald et al. identify the magnetic ordering transition using neutron diffraction by encoding physical details of the measurement (e.g., hysteresis, appropriate parameter distributions) and further by automatically selecting from a set of analytical models for the final analysis.^88^ Each of these studies presents lessons that should be adaptable to the process of phase mapping of polymer materials. As discussed above, the incorporation of domain knowledge into ML models greatly increases the accuracy of the model and reduces the data needed to achieve that accuracy. The polymers community has a rich array of theories and models that can be used to enhance autonomous agents and phase-mapping tasks.

As outlined in Table 1, one of the key challenges of applying autonomous techniques is the construction, operation, and maintenance of the robotic platform itself. In addition to domain-specific and ML expertise, building an autonomous platform requires a confluence of skills (machining, fabrication, electronic design, embedded software, robotics) that do not commonly overlap with polymers research groups. In a recent perspective, MacLeod et al. write about the importance and challenges of building flexible, multiuse robotic platforms.^189^ In a separate work, they also demonstrate how flexible platforms can identify the temperature–conductivity Pareto front in metallic thin films.^190^

Outside of autonomous, there are also several ML developments from the greater materials community worth highlighting. Gomes et al. have developed an unsupervised background subtraction methodology based on NMF techniques and have applied it to XRD and Raman spectroscopy data sets of metal-oxide samples.^191^ The fact that this method is unsupervised means it does not require examples of background spectra and can be applied to unlabeled data. Furthermore, while demonstrated on XRD and Raman measurements, the construction of the approach is sufficiently general such that it should be applicable to many other measurements and kinds of materials. In their recent paper, Liang et al. develop a ML algorithm to automatically process images from reflection high-energy electron diffraction measurements of epitaxial thin films of iron oxides.^192^ The authors combine image segmentation, transfer learning, unsupervised clustering, and traditional mathematical analyses in order to extract diffraction peaks from the images, process them, and cluster them into phases. The work is a nice demonstration of how many individual ML methods can be brought together to automate a tedious analysis that is traditionally done by hand.

## Summary

The communities of polymer physics and chemistry are working to realize the promise of machine learning and, along the way, they are discovering and addressing key challenges arising from the unique nature of polymer materials. Researchers are developing methods to represent the statistical nature of polymer structure and encode polymer domain knowledge in machine learning models. Autonomous experimentation techniques, which are far more established in other materials and chemistry fields, are being adopted and extended by polymers researchers to synthesize, characterize, and formulate materials more rapidly, with higher resolution, and at reduced cost. Partly in an effort to tackle problem of having few, small, poorly annotated data sets, efforts to use transfer learning which leverage larger data sets from others fields are starting to bear fruit. Overall, the future of machine learning is bright for polymer materials, but there is still much work to be done.

In order to unlock the true potential of machine learning, the polymer community must become more collaborative. High-quality open data, codebases, and benchmarks are essential to continued forward progress. Shared data sets must be provided with robust metadata, in accordance with FAIR data principles. Both analysis and production codes should be written with shareability, maintainability, and reuse in mind. Benchmarks should be created that provide a “ground-truth” that researchers can use to validate their methods and to make claims of improvement against. These practices will aid the adoption and advancement of machine learning within the polymer community, thereby accelerating materials and knowledge discovery in future studies.

## References

[ref1] Ramesh A.; Dhariwal P.; Nichol A.; Chu C.; Chen M. Hierarchical Text-Conditional Image Generation with CLIP Latents. arXiv 2022, https://arxiv.org/abs/2204.06125 (submitted 2022-04-13; accessed 2022-12-05).

[ref2] Yang Y.; Yuan Y.; Zhang G.; Wang H.; Chen Y.-C.; Liu Y.; Tarolli C. G.; Crepeau D.; Bukartyk J.; Junna M. R.; Videnovic A.; Ellis T. D.; Lipford M. C.; Dorsey R.; Katabi D. Artificial intelligence-enabled detection and assessment of Parkinson’s disease using nocturnal breathing signals. Nature medicine 2022, 28, 2207–2215. 10.1038/s41591-022-01932-x. PMC9556299 35995955

[ref3] Mittal A.; et al. Artificial intelligence uncovers carcinogenic human metabolites. Nat. Chem. Biol. 2022, 18, 1204–1213. 10.1038/s41589-022-01110-7. 35953549

[ref4] Jumper J.; Evans R.; Pritzel A.; Green T.; Figurnov M.; Ronneberger O.; Tunyasuvunakool K.; Bates R.; Žídek A.; Potapenko A.; et al. Highly accurate protein structure prediction with AlphaFold. Nature 2021, 596, 583–589. 10.1038/s41586-021-03819-2. 34265844 PMC8371605

[ref5] Morgan D.; Jacobs R. Opportunities and challenges for machine learning in materials science. Annu. Rev. Mater. Res. 2020, 50, 71–103. 10.1146/annurev-matsci-070218-010015.

[ref6] Batra R.; Song L.; Ramprasad R. Emerging materials intelligence ecosystems propelled by machine learning. Nature Reviews Materials 2021, 6, 655–678. 10.1038/s41578-020-00255-y.

[ref7] Pilania G. Machine learning in materials science: From explainable predictions to autonomous design. Comput. Mater. Sci. 2021, 193, 110360 10.1016/j.commatsci.2021.110360.

[ref8] Kadulkar S.; Sherman Z. M.; Ganesan V.; Truskett T. M. Machine learning-assisted design of material properties. Annu. Rev. Chem. Biomol. Eng. 2022, 13, 235–254. 10.1146/annurev-chembioeng-092220-024340. 35300515

[ref9] Audus D. J.; de Pablo J. J. Polymer informatics: Opportunities and challenges. ACS macro letters 2017, 6, 1078–1082. 10.1021/acsmacrolett.7b00228. 29201535 PMC5702941

[ref10] Peerless J. S.; Milliken N. J.; Oweida T. J.; Manning M. D.; Yingling Y. G. Soft matter informatics: current progress and challenges. Advanced Theory and Simulations 2019, 2, 1800129 10.1002/adts.201800129.

[ref11] Jackson N. E.; Webb M. A.; de Pablo J. J. Recent advances in machine learning towards multiscale soft materials design. Current Opinion in Chemical Engineering 2019, 23, 106–114. 10.1016/j.coche.2019.03.005.

[ref12] Wu S.; Yamada H.; Hayashi Y.; Zamengo M.; Yoshida R. Potentials and challenges of polymer informatics: exploiting machine learning for polymer design. arXiv 2010, https://arxiv.org/abs/2010.07683 (submitted date 2020-10-5; accessed 2022-12-05).

[ref13] Sha W.; Li Y.; Tang S.; Tian J.; Zhao Y.; Guo Y.; Zhang W.; Zhang X.; Lu S.; Cao Y.-C.; et al. Machine learning in polymer informatics. InfoMat 2021, 3, 353–361. 10.1002/inf2.12167.

[ref14] Chen L.; Pilania G.; Batra R.; Huan T. D.; Kim C.; Kuenneth C.; Ramprasad R. Polymer informatics: Current status and critical next steps. Materials Science and Engineering: R: Reports 2021, 144, 100595 10.1016/j.mser.2020.100595.

[ref15] Sattari K.; Xie Y.; Lin J. Data-driven algorithms for inverse design of polymers. Soft Matter 2021, 17, 7607–7622. 10.1039/D1SM00725D. 34397078

[ref16] Gormley A. J.; Webb M. A. Machine learning in combinatorial polymer chemistry. Nature Reviews Materials 2021, 6, 642–644. 10.1038/s41578-021-00282-3. PMC8356908 34394961

[ref17] Reinhart W. F.; Statt A. Opportunities and challenges for inverse design of nanostructures with sequence defined macromolecules. Accounts of Materials Research 2021, 2, 697–700. 10.1021/accountsmr.1c00089.

[ref18] Patra T. K. Data-driven methods for accelerating polymer design. ACS Polymers Au 2022, 2, 8–26. 10.1021/acspolymersau.1c00035. 36855746 PMC9954355

[ref19] Cencer M. M.; Moore J. S.; Assary R. S. Machine learning for polymeric materials: an introduction. Polym. Int. 2022, 71, 537–542. 10.1002/pi.6345.

[ref20] Meyer T. A.; Ramirez C.; Tamasi M. J.; Gormley A. J. A User Guide to Machine Learning for Polymeric Biomaterials. ACS Polymers Au 2022, 10.1021/acspolymersau.2c00037. PMC10103193 37065715

[ref21] Ma R.; Luo T. PI1M: a benchmark database for polymer informatics. J. Chem. Inf. Model. 2020, 60, 4684–4690. 10.1021/acs.jcim.0c00726. 32986418

[ref22] Barnett J. W.; Bilchak C. R.; Wang Y.; Benicewicz B. C.; Murdock L. A.; Bereau T.; Kumar S. K. Designing exceptional gas-separation polymer membranes using machine learning. Science advances 2020, 6, eaaz4301 10.1126/sciadv.aaz4301. 32440545 PMC7228755

[ref23] Webb M. A.; Jackson N. E.; Gil P. S.; de Pablo J. J. Targeted sequence design within the coarse-grained polymer genome. Science advances 2020, 6, eabc6216 10.1126/sciadv.abc6216. 33087352 PMC7577717

[ref24] Kuenneth C.; Lalonde J.; Marrone B. L.; Iverson C. N.; Ramprasad R.; Pilania G. Bioplastic Design using Multitask Deep Neural Networks. Commun. Mater. 2022, 3, 96 10.1038/s43246-022-00319-2.

[ref25] Bone J. M.; Childs C. M.; Menon A.; Poczos B.; Feinberg A. W.; LeDuc P. R.; Washburn N. R. Hierarchical machine learning for high-fidelity 3D printed biopolymers. ACS Biomaterials Science & Engineering 2020, 6, 7021–7031. 10.1021/acsbiomaterials.0c00755. 33320614

[ref26] Reis M.; Gusev F.; Taylor N. G.; Chung S. H.; Verber M. D.; Lee Y. Z.; Isayev O.; Leibfarth F. A. Machine-Learning-Guided Discovery of F-19 MRI Agents Enabled by Automated Copolymer Synthesis. J. Am. Chem. Soc. 2021, 143, 17677–17689. 10.1021/jacs.1c08181. 34637304 PMC10833148

[ref27] Yamada H.; Liu C.; Wu S.; Koyama Y.; Ju S.; Shiomi J.; Morikawa J.; Yoshida R. Predicting materials properties with little data using shotgun transfer learning. ACS central science 2019, 5, 1717–1730. 10.1021/acscentsci.9b00804. 31660440 PMC6813555

[ref28] Dhamankar S.; Webb M. A. Chemically specific coarse-graining of polymers: Methods and prospects. J. Polym. Sci. 2021, 59, 2613–2643. 10.1002/pol.20210555.

[ref29] Hastie T.; Friedman J.; Tisbshirani R. The Elements of Statistical Learning: Data Mining, Inference, and Prediction; Springer, 2017.

[ref30] James G.; Witten D.; Hastie T.; Tibshirani R. An Introduction to Statistical Learning: With Applications in R, 2nd ed.; Springer, 2013.

[ref31] Goodfellow I.; Bengio Y.; Courville A. Deep Learning; MIT Press, 2016; http://www.deeplearningbook.org.

[ref32] Settles B. Active Learning; Morgan & Claypool, 2012.

[ref33] Ma R.; Zhang H.; Luo T. Exploring High Thermal Conductivity Amorphous Polymers Using Reinforcement Learning. ACS Appl. Mater. Interfaces 2022, 14, 15587–15598. 10.1021/acsami.1c23610. 35344333

[ref34] Jablonka K. M.; Jothiappan G. M.; Wang S.; Smit B.; Yoo B. Bias free multiobjective active learning for materials design and discovery. Nat. Commun. 2021, 12, 2312 10.1038/s41467-021-22437-0. 33875649 PMC8055971

[ref35] Smith S.; Using DeepSpeed and Megatron to Train Megatron-Turing NLG 530B, A Large-Scale Generative Language Model. arXiv 2022, https://arxiv.org/abs/2201.11990.

[ref36] Any identification of software, equipment, instruments, companies, or materials in this work is done so for the express purposes of completeness and understanding. Such identification does not imply recommendation or endorsement by the National Institute of Standards and Technology, nor does it imply that the materials or equipment identified are necessarily the best available for the purpose.

[ref37] Jha A.; Chandrasekaran A.; Kim C.; Ramprasad R. Impact of dataset uncertainties on machine learning model predictions: the example of polymer glass transition temperatures. Modell. Simul. Mater. Sci. Eng. 2019, 27, 024002 10.1088/1361-651X/aaf8ca.

[ref38] Wilkinson M. D.; Dumontier M.; Aalbersberg I. J.; Appleton G.; Axton M.; Baak A.; Blomberg N.; Boiten J.-W.; da Silva Santos L. B.; Bourne P. E.; et al. The FAIR Guiding Principles for scientific data management and stewardship. Scientific data 2016, 3, 160018 10.1038/sdata.2016.18. 26978244 PMC4792175

[ref39] Baudis S.; Behl M. High-Throughput and Combinatorial Approaches for the Development of Multifunctional Polymers. Macromol. Rapid Commun. 2022, 43, 2100400 10.1002/marc.202100400. 34460146

[ref40] Huan T. D.; Mannodi-Kanakkithodi A.; Kim C.; Sharma V.; Pilania G.; Ramprasad R. A polymer dataset for accelerated property prediction and design. Scientific data 2016, 3, 160012 10.1038/sdata.2016.12. 26927478 PMC4772654

[ref41] Patra T. K.; Meenakshisundaram V.; Hung J.-H.; Simmons D. S. Neural-network-biased genetic algorithms for materials design: evolutionary algorithms that learn. ACS combinatorial science 2017, 19, 96–107. 10.1021/acscombsci.6b00136. 27997791

[ref42] Afzal M. A. F.; Browning A. R.; Goldberg A.; Halls M. D.; Gavartin J. L.; Morisato T.; Hughes T. F.; Giesen D. J.; Goose J. E. High-throughput molecular dynamics simulations and validation of thermophysical properties of polymers for various applications. ACS Applied Polymer Materials 2021, 3, 620–630. 10.1021/acsapm.0c00524.

[ref43] Hung J.-H.; Patra T. K.; Simmons D. S. Forecasting the experimental glass transition from short time relaxation data. J. Non-Cryst. Solids 2020, 544, 120205 10.1016/j.jnoncrysol.2020.120205.

[ref44] Ma R.; Zhang H.; Xu J.; Hayashi Y.; Yoshida R.; Shiomi J.; Luo T. Machine Learning-Assisted Exploration of Thermally Conductive Polymers Based on High-Throughput Molecular Dynamics Simulations. Mater. Today Phys. 2022, 28, 100850 10.1016/j.mtphys.2022.100850.

[ref45] Tao L.; Varshney V.; Li Y. Benchmarking Machine Learning Models for Polymer Informatics: An Example of Glass Transition Temperature. J. Chem. Inf. Model. 2021, 61, 5395–5413. 10.1021/acs.jcim.1c01031. 34662106

[ref46] Hong Z.; Tchoua R.; Chard K.; Foster I. SciNER: extracting named entities from scientific literature 2020, 12138, 308–321. 10.1007/978-3-030-50417-5_23.

[ref47] Shetty P.; Ramprasad R. Machine-Guided Polymer Knowledge Extraction Using Natural Language Processing: The Example of Named Entity Normalization. J. Chem. Inf. Model. 2021, 61, 5377–5385. 10.1021/acs.jcim.1c00554. 34752101

[ref48] Tchoua R. B.; Chard K.; Audus D. J.; Ward L. T.; Lequieu J.; De Pablo J. J.; Foster I. T. Towards a hybrid human-computer scientific information extraction pipeline. 2017 IEEE 13th international conference on e-Science (e-Science) 2017, 109–118.

[ref49] Oka H.; Yoshizawa A.; Shindo H.; Matsumoto Y.; Ishii M. Machine extraction of polymer data from tables using XML versions of scientific articles. Science and Technology of Advanced Materials: Methods 2021, 1, 12–23. 10.1080/27660400.2021.1899456.

[ref50] Shetty P.; Ramprasad R. Automated knowledge extraction from polymer literature using natural language processing. Iscience 2021, 24, 101922 10.1016/j.isci.2020.101922. 33458607 PMC7797509

[ref51] Lin C.; Wang P.-H.; Hsiao Y.; Chan Y.-T.; Engler A. C.; Pitera J. W.; Sanders D. P.; Cheng J.; Tseng Y. J. Essential step toward mining big polymer data: polyname2structure, mapping polymer names to structures. ACS Applied Polymer Materials 2020, 2, 3107–3113. 10.1021/acsapm.0c00273.

[ref52] MaterialsMine; https://materialsmine.org/nm#/ (accessed 2022-08-15).

[ref53] Brinson L. C.; Deagen M.; Chen W.; McCusker J.; McGuinness D. L.; Schadler L. S.; Palmeri M.; Ghumman U.; Lin A.; Hu B. Polymer nanocomposite data: curation, frameworks, access, and potential for discovery and design. ACS Macro Lett. 2020, 9, 1086–1094. 10.1021/acsmacrolett.0c00264. 35653211

[ref54] A Community Resource for Innovation in Polymer Technology; https://criptapp.org/ (accessed 2023-01-13). 10.1021/acscentsci.3c00011 PMC10037456 36968543

[ref55] Ting J. M.; Lipscomb C. E. Launching a materials informatics initiative for industrial applications in materials science, chemistry, and engineering. Pure Appl. Chem. 2022, 94, 637 10.1515/pac-2022-0101.

[ref56] RDKit: Open-source cheminformatics; https://www.rdkit.org (accessed 2022-08-15).

[ref57] Lin T.-S.; Coley C. W.; Mochigase H.; Beech H. K.; Wang W.; Wang Z.; Woods E.; Craig S. L.; Johnson J. A.; Kalow J. A.; et al. BigSMILES: a structurally-based line notation for describing macromolecules. ACS central science 2019, 5, 1523–1531. 10.1021/acscentsci.9b00476. 31572779 PMC6764162

[ref58] Guo M.; Shou W.; Makatura L.; Erps T.; Foshey M.; Matusik W. Polygrammar: Grammar for Digital Polymer Representation and Generation. Advanced Science 2022, 9, 2101864 10.1002/advs.202101864. 35678650 PMC9376847

[ref59] Kuenneth C.; Schertzer W.; Ramprasad R. Copolymer informatics with multitask deep neural networks. Macromolecules 2021, 54, 5957–5961. 10.1021/acs.macromol.1c00728.

[ref60] Bhattacharya D.; Kleeblatt D. C.; Statt A.; Reinhart W. F. Predicting aggregate morphology of sequence-defined macromolecules with recurrent neural networks. Soft Matter 2022, 18, 5037–5051. 10.1039/D2SM00452F. 35748651

[ref61] Patel R. A.; Borca C. H.; Webb M. A. Featurization strategies for polymer sequence or composition design by machine learning. Mol. Syst. Des. Eng. 2022, 7, 661–676. 10.1039/D1ME00160D.

[ref62] Aldeghi M.; Coley C. W. A graph representation of molecular ensembles for polymer property prediction. Chem. Sci. 2022, 13, 10486–10498. 10.1039/D2SC02839E. 36277616 PMC9473492

[ref63] Cao L.; Russo D.; Felton K.; Salley D.; Sharma A.; Keenan G.; Mauer W.; Gao H.; Cronin L.; Lapkin A. A. Optimization of Formulations Using Robotic Experiments Driven by. Machine Learning DoE. Cell Reports Physical Science 2021, 2, 100295 10.1016/j.xcrp.2020.100295.

[ref65] Bradford E.; Schweidtmann A. M.; Lapkin A. Efficient multiobjective optimization employing Gaussian processes, spectral sampling and a genetic algorithm. Journal of Global Optimization 2018, 71, 407–438. 10.1007/s10898-018-0609-2.

[ref66] Ethier J. G.; Casukhela R. K.; Latimer J. J.; Jacobsen M. D.; Rasin B.; Gupta M. K.; Baldwin L. A.; Vaia R. A. Predicting Phase Behavior of Linear Polymers in Solution Using. Machine Learning. Macromolecules 2022, 55, 2691–2702. 10.1021/acs.macromol.2c00245.

[ref67] Kim C.; Chandrasekaran A.; Jha A.; Ramprasad R. Active-learning and materials design: the example of high glass transition temperature polymers. Mrs Communications 2019, 9, 860–866. 10.1557/mrc.2019.78.

[ref68] Zhao S.; Cai T.; Zhang L.; Li W.; Lin J. Autonomous Construction of Phase Diagrams of Block Copolymers by Theory-Assisted Active. Machine Learning. ACS Macro Letters 2021, 10, 598–602. 10.1021/acsmacrolett.1c00133. 35570770

[ref69] Arora A.; Lin T.-S.; Rebello N. J.; Av-Ron S. H.; Mochigase H.; Olsen B. D. Random forest predictor for diblock copolymer phase behavior. ACS Macro Lett. 2021, 10, 1339–1345. 10.1021/acsmacrolett.1c00521. 35549019

[ref70] Ristoski P.; Zubarev D. Y.; Gentile A. L.; Park N.; Sanders D.; Gruhl D.; Kato L.; Welch S. Expert-in-the-Loop AI for Polymer Discovery. Proceedings of the 29th ACM International Conference on Information & Knowledge Management; New York, NY, USA, 2020; p 2701–2708.

[ref71] Wheatle B. K.; Fuentes E. F.; Lynd N. A.; Ganesan V. Design of polymer blend electrolytes through a machine learning approach. Macromolecules 2020, 53, 9449–9459. 10.1021/acs.macromol.0c01547.

[ref72] Pollice R.; dos Passos Gomes G.; Aldeghi M.; Hickman R. J.; Krenn M.; Lavigne C.; Lindner-D’Addario M.; Nigam A.; Ser C. T.; Yao Z.; Aspuru-Guzik A. Data-Driven Strategies for Accelerated Materials Design. Acc. Chem. Res. 2021, 54, 849–860. 10.1021/acs.accounts.0c00785. 33528245 PMC7893702

[ref73] Roch L. M.; Häse F.; Kreisbeck C.; Tamayo-Mendoza T.; Yunker L. P. E.; Hein J. E.; Aspuru-Guzik A. ChemOS: An orchestration software to democratize autonomous discovery. PLoS One 2020, 15, e0229862 10.1371/journal.pone.0229862. 32298284 PMC7161969

[ref74] Flores-Leonar M. M.; Mejía-Mendoza L. M.; Aguilar-Granda A.; Sanchez-Lengeling B.; Tribukait H.; Amador-Bedolla C.; Aspuru-Guzik A. Materials Acceleration Platforms: On the way to autonomous experimentation. Current Opinion in Green and Sustainable Chemistry 2020, 25, 100370 10.1016/j.cogsc.2020.100370.

[ref75] Volk A. A.; Epps R. W.; Abolhasani M. Accelerated Development of Colloidal Nanomaterials Enabled by Modular Microfluidic Reactors: Toward Autonomous Robotic Experimentation. Adv. Mater. 2021, 33, 2004495 10.1002/adma.202004495. 33289177

[ref76] Volk A. A.; Abolhasani M. Autonomous flow reactors for discovery and invention. Trends in Chemistry 2021, 3, 519–522. 10.1016/j.trechm.2021.04.001.

[ref77] Epps R. W.; Abolhasani M. Modern nanoscience: Convergence of AI, robotics, and colloidal synthesis. Applied Physics Reviews 2021, 8, 041316 10.1063/5.0061799.

[ref78] Vaddi K.; Chiang H. T.; Pozzo L. D. Autonomous retrosynthesis of gold nanoparticles via spectral shape matching. Digital Discovery 2022, 1, 502–510. 10.1039/D2DD00025C.

[ref79] Upadhya R.; Kosuri S.; Tamasi M.; Meyer T. A.; Atta S.; Webb M. A.; Gormley A. J. Automation and data-driven design of polymer therapeutics. Adv. Drug Delivery Rev. 2021, 171, 1–28. 10.1016/j.addr.2020.11.009. PMC8127395 33242537

[ref80] Tamasi M. J.; Patel R. A.; Borca C. H.; Kosuri S.; Mugnier H.; Upadhya R.; Murthy N. S.; Webb M. A.; Gormley A. J. Machine Learning on a Robotic Platform for the Design of Polymer-Protein Hybrids. Adv. Mater. 2022, 34, 2201809 10.1002/adma.202201809. PMC9339531 35593444

[ref81] Knox S. T.; Parkinson S. J.; Wilding C. Y. P.; Bourne R. A.; Warren N. J. Autonomous polymer synthesis delivered by multi-objective closed-loop optimisation. Polym. Chem. 2022, 13, 1576–1585. 10.1039/D2PY00040G.

[ref82] Chen C.; Richter F.; Zhang J.; Guerrero-Sanchez C.; Traeger A.; Schubert U. S.; Feng A.; Thang S. H. Synthesis of functional miktoarm star polymers in an automated parallel synthesizer. Eur. Polym. J. 2021, 160, 110777 10.1016/j.eurpolymj.2021.110777.

[ref83] Tamasi M.; Kosuri S.; DiStefano J.; Chapman R.; Gormley A. J. Automation of Controlled/Living Radical Polymerization. Advanced Intelligent Systems 2020, 2, 1900126 10.1002/aisy.201900126. 35586369 PMC9113399

[ref84] Van Herck J.; Abeysekera I.; Buckinx A.-L.; Cai K.; Hooker J.; Thakur K.; Van de Reydt E.; Voorter P.-J.; Wyers D.; Junkers T. Operator-independent high-throughput polymerization screening based on automated inline NMR and online SEC. Digital Discovery 2022, 1, 519–526. 10.1039/D2DD00035K.

[ref85] Langner S.; Hase F.; Perea J. D.; Stubhan T.; Hauch J.; Roch L. M.; Heumueller T.; Aspuru-Guzik A.; Brabec C. J. Beyond Ternary OPV: High-Throughput Experimentation and Self-Driving Laboratories Optimize Multicomponent Systems. Adv. Mater. 2020, 32, 1907801 10.1002/adma.201907801. 32049386

[ref86] Martineau R. L.; Bayles A. V.; Hung C.-S.; Reyes K. G.; Helgeson M. E.; Gupta M. K. Engineering Gelation Kinetics in Living Silk Hydrogels by Differential Dynamic Microscopy Microrheology and Machine Learning. Advanced Biology 2022, 6, 2101070 10.1002/adbi.202101070. 34811969

[ref87] Rodriguez J.; Politi M.; Adler S.; Beck D.; Pozzo L. High-throughput and data driven strategies for the design of deep-eutectic solvent electrolytes. Molecular Systems Design & Engineering 2022, 7, 933–949. 10.1039/D2ME00050D.

[ref88] McDannald A.; Frontzek M.; Savici A. T.; Doucet M.; Rodriguez E. E.; Meuse K.; Opsahl-Ong J.; Samarov D.; Takeuchi I.; Ratcliff W.; Kusne A. G. On-the-fly autonomous control of neutron diffraction via physics-informed Bayesian active learning. Applied Physics Reviews 2022, 9, 021408 10.1063/5.0082956.

[ref89] Budd S.; Robinson E. C.; Kainz B. A survey on active learning and human-in-the-loop deep learning for medical image analysis. Medical Image Analysis 2021, 71, 102062 10.1016/j.media.2021.102062. 33901992

[ref90] Stach E.; et al. Autonomous experimentation systems for materials development: A community perspective. Matter 2021, 4, 2702–2726. 10.1016/j.matt.2021.06.036.

[ref91] Hensman J.; Fusi N.; Lawrence N. D. Gaussian Processes for Big Data. AUAI 2013, 282–290. 10.5555/3023638.3023667.

[ref92] Feurer M.; Klein A.; Eggensperger K.; Springenberg J.; Blum M.; Hutter F. Machine Learning. Advances in Neural Information Processing Systems. Advances in Neural Information Processing Systems; Curran Associates, Inc., 2015.

[ref93] Feurer M.; Klein A.; Eggensperger K.; Springenberg J.; Blum M.; Hutter F. https://github.com/automl/auto-sklearn (accessed 2022-08-15).

[ref94] Xu J., Darancet P. T. Polybot; https://www.anl.gov/cnm/polybot (accessed 2022-11-26).

[ref95] Beaucage P.; Martin T. Autonomous Formulation Lab; https://www.nist.gov/ncnr/ncnr-facility-upgrades/autonomous-formulation-lab-afl (accessed 2022-11-26).

[ref96] Barredo Arrieta A.; Diaz-Rodriguez N.; Del Ser J.; Bennetot A.; Tabik S.; Barbado A.; Garcia S.; Gil-Lopez S.; Molina D.; Benjamins R.; et al. Explainable Artificial Intelligence (XAI): Concepts, taxonomies, opportunities and challenges toward responsible AI. Information Fusion 2020, 58, 82–115. 10.1016/j.inffus.2019.12.012.

[ref97] Tibshirani R. Regression shrinkage and selection via the lasso. Journal of the Royal Statistical Society: Series B (Methodological) 1996, 58, 267–288. 10.1111/j.2517-6161.1996.tb02080.x.

[ref98] Murphy K. P. Machine Learning: A Probabilistic Perspective; MIT Press, 2012.

[ref99] Ouyang R.; Curtarolo S.; Ahmetcik E.; Scheffler M.; Ghiringhelli L. M. SISSO: A compressed-sensing method for identifying the best low-dimensional descriptor in an immensity of offered candidates. Physical Review Materials 2018, 2, 083802 10.1103/PhysRevMaterials.2.083802.

[ref100] Pilania G.; Iverson C. N.; Lookman T.; Marrone B. L. Machine-learning-based predictive modeling of glass transition temperatures: A case of polyhydroxyalkanoate homopolymers and copolymers. J. Chem. Inf. Model. 2019, 59, 5013–5025. 10.1021/acs.jcim.9b00807. 31697891

[ref101] Augusto D. A.; Barbosa H. J. Symbolic regression via genetic programming. Proceedings of the Sixth Brazilian Symposium on Neural Networks; IEEE, 2000; Vol. 1, pp 173–178.

[ref102] Wang Y.; Wagner N.; Rondinelli J. M. Symbolic regression in materials science. MRS Commun. 2019, 9, 793–805. 10.1557/mrc.2019.85.

[ref103] Sun S.; Ouyang R.; Zhang B.; Zhang T.-Y. Data-driven discovery of formulas by symbolic regression. MRS Bull. 2019, 44, 559–564. 10.1557/mrs.2019.156.

[ref104] Lou Y.; Caruana R.; Gehrke J.; Hooker G. Accurate intelligible models with pairwise interactions. Proceedings of the 19th ACM SIGKDD international conference on Knowledge discovery and data mining 2013, 623–631. 10.1145/2487575.2487579.

[ref105] Lundberg S. M.; Lee S.-I. A unified approach to interpreting model predictions. NIPS'17: Proceedings of the 31st International Conference on Neural Information Processing Systems; Curran Associates Inc.: Red Hook, NY, 2017; Vol. 30.

[ref106] Ribeiro M. T.; Singh S.; Guestrin C.; Why Should I. Why Should I Trust You?”: Explaining the Predictions of Any Classifier. Proceedings of the 22nd ACM SIGKDD International Conference on Knowledge Discovery and Data Mining; New York, NY, USA, 2016; pp 1135–1144; 10.1145/2939672.2939778.

[ref107] Yang J.; Tao L.; He J.; McCutcheon J.; Li Y. Discovery of Innovative Polymers for Next-Generation Gas-Separation Membranes using Interpretable Machine Learning. ChemRxiv 2021, https://chemrxiv.org/engage/chemrxiv/article-details/61c59c9d9efae73b4f255c59 (submitted 2021-12-26; accessed 2022-12-05). 10.1126/sciadv.abn9545 PMC9299556 35857839

[ref108] Kuenneth C.; Rajan A. C.; Tran H.; Chen L.; Kim C.; Ramprasad R. Polymer informatics with multi-task learning. Patterns 2021, 2, 100238 10.1016/j.patter.2021.100238. 33982028 PMC8085610

[ref109] Amamoto Y.; Kikutake H.; Kojio K.; Takahara A.; Terayama K. Visualization of judgment regions in convolutional neural networks for X-ray diffraction and scattering images of aliphatic polyesters. Polym. J. 2021, 53, 1269–1279. 10.1038/s41428-021-00531-w.

[ref110] Bejagam K. K.; Lalonde J.; Iverson C. N.; Marrone B. L.; Pilania G. Machine Learning for Melting Temperature Predictions and Design in Polyhydroxyalkanoate-Based Biopolymers. J. Phys. Chem. B 2022, 126, 934–945. 10.1021/acs.jpcb.1c08354. 35072485

[ref111] Lahat D.; Adali T.; Jutten C. Multimodal data fusion: an overview of methods, challenges, and prospects. Proceedings of the IEEE 2015, 103, 1449–1477. 10.1109/JPROC.2015.2460697.

[ref112] Meng T.; Jing X.; Yan Z.; Pedrycz W. A survey on machine learning for data fusion. Information Fusion 2020, 57, 115–129. 10.1016/j.inffus.2019.12.001.

[ref113] Wang Y.; Zhang M.; Lin A.; Iyer A.; Prasad A. S.; Li X.; Zhang Y.; Schadler L. S.; Chen W.; Brinson L. C. Mining structure-property relationships in polymer nanocomposites using data driven finite element analysis and multi-task convolutional neural networks. Molecular Systems Design & Engineering 2020, 5, 962–975. 10.1039/D0ME00020E.

[ref114] Bonettini N.; Gonano C. A.; Bestagini P.; Marcon M.; Garavelli B.; Tubaro S. Multitask learning for denoising and analysis of X-ray polymer acquisitions. IEEE Xplore 2021, 1391–1395. 10.23919/EUSIPCO54536.2021.9616220.

[ref115] Venkatram S.; Batra R.; Chen L.; Kim C.; Shelton M.; Ramprasad R. Predicting crystallization tendency of polymers using multifidelity information fusion and machine learning. J. Phys. Chem. B 2020, 124, 6046–6054. 10.1021/acs.jpcb.0c01865. 32539396

[ref116] Pan S. J.; Yang Q. A survey on transfer learning. IEEE Transactions on knowledge and data engineering 2010, 22, 1345–1359. 10.1109/TKDE.2009.191.

[ref117] Li X.; Zhang Y.; Zhao H.; Burkhart C.; Brinson L. C.; Chen W. A transfer learning approach for microstructure reconstruction and structure-property predictions. Sci. Rep. 2018, 8, 13461 10.1038/s41598-018-31571-7. 30194426 PMC6128837

[ref118] Wu S.; Kondo Y.; Kakimoto M.-a.; Yang B.; Yamada H.; Kuwajima I.; Lambard G.; Hongo K.; Xu Y.; Shiomi J.; et al. Machine-learning-assisted discovery of polymers with high thermal conductivity using a molecular design algorithm. Npj Computational Materials 2019, 5, 1–11. 10.1038/s41524-019-0203-2.

[ref119] Tsubaki M.; Mizoguchi T. Quantum deep descriptor: Physically informed transfer learning from small molecules to polymers. J. Chem. Theory Comput. 2021, 17, 7814–7821. 10.1021/acs.jctc.1c00568. 34846893

[ref120] Munshi J.; Chen W.; Chien T.; Balasubramanian G. Transfer learned designer polymers for organic solar cells. J. Chem. Inf. Model. 2021, 61, 134–142. 10.1021/acs.jcim.0c01157. 33410685

[ref121] Lu S.; Montz B.; Emrick T.; Jayaraman A. Semi-supervised machine learning model for analysis of nanowire morphologies from transmission electron microscopy images. arXiv 2022, https://arxiv.org/abs/2203.13875 (submitted 2022-09-27; accessed 2022-12-05).

[ref122] Kennedy M. C.; O’Hagan A. Predicting the output from a complex computer code when fast approximations are available. Biometrika 2000, 87, 1–13. 10.1093/biomet/87.1.1.

[ref123] Patra A.; Batra R.; Chandrasekaran A.; Kim C.; Huan T. D.; Ramprasad R. A multi-fidelity information-fusion approach to machine learn and predict polymer bandgap. Comput. Mater. Sci. 2020, 172, 109286 10.1016/j.commatsci.2019.109286.

[ref124] Wagner N.; Rondinelli J. M. Theory-guided machine learning in materials science. Frontiers in Materials 2016, 3, 28 10.3389/fmats.2016.00028.

[ref125] Karpatne A.; Atluri G.; Faghmous J. H.; Steinbach M.; Banerjee A.; Ganguly A.; Shekhar S.; Samatova N.; Kumar V. Theory-guided data science: A new paradigm for scientific discovery from data. IEEE Transactions on knowledge and data engineering 2017, 29, 2318–2331. 10.1109/TKDE.2017.2720168.

[ref126] Childs C. M.; Washburn N. R. Embedding domain knowledge for machine learning of complex material systems. MRS Commun. 2019, 9, 806–820. 10.1557/mrc.2019.90.

[ref127] Battaglia P. W.; Relational inductive biases, deep learning, and graph networks. arXiv 2018, https://arxiv.org/abs/1806.01261 (submitted 2018-10-17; accessed 2022-12-05).

[ref128] Pugar J. A.; Childs C. M.; Huang C.; Haider K. W.; Washburn N. R. Elucidating the Physicochemical Basis of the Glass Transition Temperature in Linear Polyurethane Elastomers with Machine Learning. J. Phys. Chem. B 2020, 124, 9722–9733. 10.1021/acs.jpcb.0c06439. 32898420

[ref129] Xuan Y.; Delaney K. T.; Ceniceros H. D.; Fredrickson G. H. Deep learning and self-consistent field theory: A path towards accelerating polymer phase discovery. J. Comput. Phys. 2021, 443, 110519 10.1016/j.jcp.2021.110519.

[ref130] Menon A.; Gupta C.; Perkins K. M.; DeCost B. L.; Budwal N.; Rios R. T.; Zhang K.; Póczos B.; Washburn N. R. Elucidating multi-physics interactions in suspensions for the design of polymeric dispersants: a hierarchical machine learning approach. Molecular Systems Design & Engineering 2017, 2, 263–273. 10.1039/C7ME00027H.

[ref131] Audus D. J.; McDannald A.; DeCost B. Leveraging Theory for Enhanced. Machine Learning. ACS Macro Letters 2022, 11, 1117–1122. 10.1021/acsmacrolett.2c00369. 36018715 PMC9912311

[ref132] Batra R.; Dai H.; Huan T. D.; Chen L.; Kim C.; Gutekunst W. R.; Song L.; Ramprasad R. Polymers for extreme conditions designed using syntax-directed variational autoencoders. Chem. Mater. 2020, 32, 10489–10500. 10.1021/acs.chemmater.0c03332.

[ref133] Hiraide K.; Hirayama K.; Endo K.; Muramatsu M. Application of deep learning to inverse design of phase separation structure in polymer alloy. Comput. Mater. Sci. 2021, 190, 110278 10.1016/j.commatsci.2021.110278.

[ref134] Archibald R. K.; Doucet M.; Johnston T.; Young S. R.; Yang E.; Heller W. T. Classifying and analyzing small-angle scattering data using weighted k nearest neighbors machine learning techniques. J. Appl. Crystallogr. 2020, 53, 326–334. 10.1107/S1600576720000552.

[ref135] Model Functions from SasView Package. https://www.sasview.org/docs/user/qtgui/Perspectives/Fitting/models/index.html (accessed 2022-11-26).

[ref136] Roe R.-J. Methods of X-ray and neutron scattering in polymer science; Topics in Polymer Science; Oxford University Press: New York, NY, 2000.

[ref137] Do C.; Chen W.-R.; Lee S. Small Angle Scattering Data Analysis Assisted by. Machine Learning Methods. MRS Advances 2020, 5, 1577–1584. 10.1557/adv.2020.130.

[ref138] Politi M.; Moeez A.; Beck D.; Adler S.; Pozzo L. HARDy: Handling Arbitrary Recognition of Data in Python. Journal of Open Source Software 2022, 7, 3829 10.21105/joss.03829.

[ref139] Beltran-Villegas D. J.; Wessels M. G.; Lee J. Y.; Song Y.; Wooley K. L.; Pochan D. J.; Jayaraman A. Computational Reverse-Engineering Analysis for Scattering Experiments on Amphiphilic Block Polymer Solutions. J. Am. Chem. Soc. 2019, 141, 14916–14930. 10.1021/jacs.9b08028. 31497951

[ref140] Wessels M. G.; Jayaraman A. Computational reverse-engineering analysis of scattering experiments (CREASE) on amphiphilic block polymer solutions: cylindrical and fibrillar assembly. Macromolecules 2021, 54, 783–796. 10.1021/acs.macromol.0c02265.

[ref141] Heil C. M.; Patil A.; Dhinojwala A.; Jayaraman A. Computational Reverse-Engineering Analysis for Scattering Experiments (CREASE) with. Machine Learning Enhancement to Determine Structure of Nanoparticle Mixtures and Solutions. ACS central science 2022, 8, 996–1007. 10.1021/acscentsci.2c00382. 35912348 PMC9335921

[ref142] Kusaba M.; Liu C.; Koyama Y.; Terakura K.; Yoshida R. Recreation of the periodic table with an unsupervised machine learning algorithm. Sci. Rep. 2021, 11, 4780 10.1038/s41598-021-81850-z. 33637773 PMC7910619

[ref143] Statt A.; Kleeblatt D. C.; Reinhart W. F. Unsupervised learning of sequence-specific aggregation behavior for a model copolymer. Soft Matter 2021, 17, 7697–7707. 10.1039/D1SM01012C. 34350929

[ref144] Xu X.; Wei Q.; Li H.; Wang Y.; Chen Y.; Jiang Y. Recognition of polymer configurations by unsupervised learning. Phys. Rev. E 2019, 99, 043307 10.1103/PhysRevE.99.043307. 31108670

[ref145] Parker Q.; Perera D.; Li Y. W.; Vogel T. Supervised and unsupervised machine learning of structural phases of polymers adsorbed to nanowires. Phys. Rev. E 2022, 105, 035304 10.1103/PhysRevE.105.035304. 35428161

[ref146] McInnes L.; Healy J.; Melville J. UMAP: Uniform Manifold Approximation and Projection for Dimension Reduction. arXiv 2018, https://arxiv.org/abs/1802.03426 (submitted 2020-09-18; accessed 2022-12-05).

[ref147] Cubuk E. D.; Ivancic R.; Schoenholz S. S.; Strickland D.; Basu A.; Davidson Z.; Fontaine J.; Hor J. L.; Huang Y.-R.; Jiang Y. Structure-property relationships from universal signatures of plasticity in disordered solids. Science 2017, 358, 1033–1037. 10.1126/science.aai8830. 29170231 PMC6047528

[ref148] UNESCO Recommendation on Open Science. 2021; https://unesdoc.unesco.org/ark:/48223/pf0000379949 (accessed 2022-11-26).

[ref150] Vicente-Saez R.; Martinez-Fuentes C. Open Science now: A systematic literature review for an integrated definition. Journal of business research 2018, 88, 428–436. 10.1016/j.jbusres.2017.12.043.

[ref151] arXiv; https://arxiv.org/ (accessed 2022-08-15).

[ref152] ChemRxiv; https://chemrxiv.org/ (accessed 2022-08-15).

[ref153] Zhao Z.; MLExchange: A web-based platform enabling exchangeable machine learning workflows for scientific studies. arXiv 2022, https://arxiv.org/abs/2208.09751 (submitted 2020-09-24; accessed 2022-12-05). 10.1109/xloop56614.2022.00007 PMC10733127 38131031

[ref154] McKiernan E. C.; Bourne P. E.; Brown C. T.; Buck S.; Kenall A.; Lin J.; Mc- Dougall D.; Nosek B. A.; Ram K.; Soderberg C. K. How open science helps researchers succeed. elife 2016, 5, e16800 10.7554/eLife.16800. 27387362 PMC4973366

[ref155] Materials Research Data Alliance (MaRDA); https://www.marda-alliance.org/ (accessed 2022-08-15).

[ref156] Blaiszik B.; Chard K.; Pruyne J.; Ananthakrishnan R.; Tuecke S.; Foster I. The materials data facility: data services to advance materials science research. Jom 2016, 68, 2045–2052. 10.1007/s11837-016-2001-3.

[ref157] Blaiszik B.; Ward L.; Schwarting M.; Gaff J.; Chard R.; Pike D.; Chard K.; Foster I. A data ecosystem to support machine learning in materials science. MRS Commun. 2019, 9, 1125–1133. 10.1557/mrc.2019.118.

[ref158] zenodo; https://zenodo.org/ (accessed 2022-08-15).

[ref159] figshare; https://figshare.com/ (accessed 2022-08-15).

[ref160] Zhao H.; Li X.; Zhang Y.; Schadler L. S.; Chen W.; Brinson L. C. Perspective: NanoMine: A material genome approach for polymer nanocomposites analysis and design. APL Materials 2016, 4, 053204 10.1063/1.4943679.

[ref161] Zhao H.; Wang Y.; Lin A.; Hu B.; Yan R.; McCusker J.; Chen W.; McGuinness D. L.; Schadler L.; Brinson L. C. NanoMine schema: An extensible data representation for polymer nanocomposites. APL Materials 2018, 6, 111108 10.1063/1.5046839.

[ref162] McCusker J. P.; Keshan N.; Rashid S.; Deagen M.; Brinson C.; McGuinness D. L. NanoMine: A knowledge graph for nanocomposite materials science. International Semantic Web Conference 2020, 12507, 144–159. 10.1007/978-3-030-62466-8_10.

[ref163] Walsh D.; Zou W.; Schneider L.; Mello R.; Deagen M.; Mysona J.; Lin T.-S.; de Pablo J.; Jensen K.; Audus D. CRIPT: A Scalable Polymer Material Data Structure. ChemRxiv 2022, https://chemrxiv.org/engage/chemrxiv/article-details/6322951abe03b232b0f6c7db (submitted 2022-09-15; accessed 2022-12-05). 10.1021/acscentsci.3c00011 PMC10037456 36968543

[ref164] Lin T.-S.; Rebello N. J.; Beech H. K.; Wang Z.; El-Zaatari B.; Lundberg D. J.; Johnson J. A.; Kalow J. A.; Craig S. L.; Olsen B. D. PolyDAT: a generic data schema for polymer characterization. J. Chem. Inf. Model. 2021, 61, 1150–1163. 10.1021/acs.jcim.1c00028. 33615783

[ref165] Pedregosa F.; et al. Scikit-learn: Machine Learning in Python. Journal of Machine Learning Research 2011, 12, 2825–2830.

[ref166] Paszke A.; In Advances in Neural Information Processing Systems 32; Wallach H., Larochelle H., Beygelzimer A., d’Alché-Buc F., Fox E., Garnett R., Eds.; Curran Associates, Inc., 2019; pp 8024–8035.

[ref167] Abadi M., TensorFlow: Large-Scale Machine Learning on Heterogeneous Systems. 2015; https://www.tensorflow.org/ (accessed 2022-11-26).

[ref168] Matthews A. G. d. G.; van der Wilk M.; Nickson T.; Fujii K.; Boukouvalas A.; León-Villagrá P.; Ghahramani Z.; Hensman J. GPflow: A Gaussian process library using TensorFlow. Journal of Machine Learning Research 2017, 18, 1–6.

[ref169] Gardner J. R.; Pleiss G.; Bindel D.; Weinberger K. Q.; Wilson A. G. GPyTorch: Blackbox Matrix-Matrix Gaussian Process Inference with GPU Acceleration. Advances in Neural Information Processing Systems 2018, 7587–7597.

[ref170] Balandat M.; Karrer B.; Jiang D. R.; Daulton S.; Letham B.; Wilson A. G.; Bakshy E. BoTorch: A Framework for Efficient Monte-Carlo Bayesian Optimization Advances in Neural Information Processing Systems 33. 2020.https://proceedings.neurips.cc/paper/2020

[ref171] Noack M.; gpCAM 2022; https://github.com/lbl-camera/gpCAM (accessed 2022-11-26).

[ref172] Meredig B. Five high-impact research areas in machine learning for materials science. Chem. Mater. 2019, 31, 9579–9581. 10.1021/acs.chemmater.9b04078.

[ref173] Wilson G. Carpentry web site. http://software-carpentry.org (accessed 2022-11-26).

[ref174] Wang A. Y.-T.; Murdock R. J.; Kauwe S. K.; Oliynyk A. O.; Gurlo A.; Brgoch J.; Persson K. A.; Sparks T. D. Machine learning for materials scientists: An introductory guide toward best practices. Chem. Mater. 2020, 32, 4954–4965. 10.1021/acs.chemmater.0c01907.

[ref175] Palizhati A.; Torrisi S. B.; Aykol M.; Suram S. K.; Hummelshoj J. S.; Montoya J. H. Agents for sequential learning using multiple-fidelity data. Sci. Rep. 2022, 12, 13 10.1038/s41598-022-08413-8. 35304496 PMC8933401

[ref176] Liang Q. H.; Gongora A. E.; Ren Z. K.; Tiihonen A.; Liu Z.; Sun S. J.; Deneault J. R.; Bash D.; Mekki-Berrada F.; Khan S. A.; Hippalgaonkar K.; Maruyama B.; Brown K. A.; Fisher J.; Buonassisi T. Benchmarking the performance of Bayesian optimization across multiple experimental materials science domains. Npj Computational Materials 2021, 7, 10 10.1038/s41524-021-00656-9.

[ref177] Protein Structure Prediction Center; https://predictioncenter.org (accessed 2022-11-23).

[ref178] Materials Genome Initiative Strategic Plan 2021; https://www.mgi.gov/sites/default/files/documents/MGI-2021-Strategic-Plan.pdf (accessed 2022-08-15).

[ref179] Montoya J. H.; Aykol M.; Anapolsky A.; Gopal C. B.; Herring P. K.; Hummelshøj J. S.; Hung L.; Kwon H.-K.; Schweigert D.; Sun S.; Suram S. K.; Torrisi S. B.; Trewartha A.; Storey B. D. Toward autonomous materials research: Recent progress and future challenges. Applied Physics Reviews 2022, 9, 011405 10.1063/5.0076324.

[ref180] Rahmanian F.; Flowers J.; Guevarra D.; Richter M.; Fichtner M.; Donnely P.; Gregoire J. M.; Stein H. S. Enabling Modular Autonomous Feedback-Loops in Materials Science through Hierarchical Experimental Laboratory Automation and Orchestration. Advanced Materials Interfaces 2022, 9, 2101987 10.1002/admi.202101987.

[ref181] Kusne A. G.; Yu H.; Wu C.; Zhang H.; Hattrick-Simpers J.; DeCost B.; Sarker S.; Oses C.; Toher C.; Curtarolo S.; et al. On-the-fly closed-loop materials discovery via Bayesian active learning. Nat. Commun. 2020, 11, 5966 10.1038/s41467-020-19597-w. 33235197 PMC7686338

[ref182] Chen D.; Bai Y. W.; Ament S.; Zhao W. T.; Guevarra D.; Zhou L.; Selman B.; van Dover R. B.; Gregoire J. M.; Gomes C. P. Automating crystal-structure phase mapping by combining deep learning with constraint reasoning. Nature Machine Intelligence 2021, 3, 812–822. 10.1038/s42256-021-00384-1.

[ref183] Gomes C. P.; Bai J.; Xue Y.; Björck J.; Rappazzo B.; Ament S.; Bernstein R.; Kong S.; Suram S. K.; van Dover R. B.; Gregoire J. M. CRYSTAL: a multi-agent AI system for automated mapping of materials crystal structures. MRS Commun. 2019, 9, 600–608. 10.1557/mrc.2019.50.

[ref184] Suram S. K.; Xue Y.; Bai J.; Le Bras R.; Rappazzo B.; Bernstein R.; Bjorck J.; Zhou L.; van Dover R. B.; Gomes C. P.; Gregoire J. M. Automated Phase Mapping with AgileFD and its Application to Light Absorber Discovery in the V-Mn-Nb Oxide System. ACS Comb. Sci. 2017, 19, 37–46. 10.1021/acscombsci.6b00153. 28064478

[ref185] Xue Y.; Bai J.; Le Bras R.; Rappazzo B.; Bernstein R.; Bjorck J.; Longpre L.; Suram S. K.; van Dover R. B.; Gregoire J.; Gomes C. P. Phase-Mapper: An AI Platform to Accelerate High Throughput Materials Discovery. 2016; https://ui.adsabs.harvard.edu/abs/2016arXiv161000689X (accessed 2022-11-26).

[ref186] Tian Y.; Lookman T.; Xue D. Z. Efficient sampling for decision making in materials discovery*. Chinese Physics B 2021, 30, 050705 10.1088/1674-1056/abf12d.

[ref187] Kusne A. G.; McDannald A.; DeCost B.; Oses C.; Toher C.; Curtarolo S.; Mehta A.; Takeuchi I. Physics in the Machine: Integrating Physical Knowledge in Autonomous Phase-Mapping. Frontiers in Physics 2022, 10, 6 10.3389/fphy.2022.815863.

[ref188] Ament S.; Amsler M.; Sutherland D. R.; Chang M. C.; Guevarra D.; Connolly A. B.; Gregoire J. M.; Thompson M. O.; Gomes C. P.; van Dover R. B. Autonomous materials synthesis via hierarchical active learning of nonequilibrium phase diagrams. Science Advances 2021, 7, 12 10.1126/sciadv.abg4930. PMC8682983 34919429

[ref189] MacLeod B. P.; Parlane F. G. L.; Brown A. K.; Hein J. E.; Berlinguette C. P. Flexible automation accelerates materials discovery. Nat. Mater. 2022, 21, 722–726. 10.1038/s41563-021-01156-3. 34907322

[ref190] MacLeod B. P.; Parlane F. G. L.; Rupnow C. C.; Dettelbach K. E.; Elliott M. S.; Morrissey T. D.; Haley T. H.; Proskurin O.; Rooney M. B.; Taherimakhsousi N.; et al. A self-driving laboratory advances the Pareto front for material properties. Nat. Commun. 2022, 13, 995 10.1038/s41467-022-28580-6. 35194074 PMC8863835

[ref191] Ament S. E.; Stein H. S.; Guevarra D.; Zhou L.; Haber J. A.; Boyd D. A.; Umehara M.; Gregoire J. M.; Gomes C. P. Multi-component background learning automates signal detection for spectroscopic data. npj Computational Materials 2019, 5, 77 10.1038/s41524-019-0213-0.

[ref192] Liang H. T.; Stanev V.; Kusne A. G.; Tsukahara Y.; Ito K.; Takahashi R.; Lippmaa M.; Takeuchi I. Application of machine learning to reflection high-energy electron diffraction images for automated structural phase mapping. Physical Review Materials 2022, 6, 063805 10.1103/PhysRevMaterials.6.063805.

